# Periodic Refresher Emails for Emergency Department Mass Casualty Incident Plans

**DOI:** 10.21980/J8C05W

**Published:** 2020-07-15

**Authors:** Jessie G Nelson, Sara Hevesi, Robert Welborn, Krista R Carlson, Benjamin Eide, Merideth Winkler, Marissa K Peterson, Elizabeth Ramey

**Affiliations:** * Regions Hospital, Department of Emergency Medicine Education Research, St. Paul, MN; ^ University of Minnesota Medical School, Department of Emergency Medicine, Minneapolis, MN; † Regions Hospital, Emergency Department, St. Paul, MN; ** Brooke Army Medical Center, San Antonio Uniformed Services Health Education Consortium, San Antonio, TX; ^^ University of Minnesota Medical School, Minneapolis, MN; †† Regions Hospital, Critical Care Research Center, St. Paul, MN; ¶ University of Minnesota, Department of Pediatrics, Minneapolis, MN

## Abstract

**Audience and Type of Curriculum:**

This mass casualty incident (MCI) curriculum is intended for use as refresher content in the months between more formal education, such as hands-on MCI training and drills. The target audience for each topic varies, but the majority of them apply to all disciplines such as direct patient care roles (emergency room technicians, nurses, paramedics, advanced practice practitioners, resident physicians, attending physicians, etc.) and emergency department clerks/coordinators. Topics intended for only one or more discipline are labeled as such. See curriculum chart or email schedule (Appendix AI) for details.

**Length of Curriculum:**

This curriculum is intended for use as weekly refresher emails spanning up to a 30-week period.

**Introduction:**

There have been an increasing number of mass casualty events occurring throughout the country in recent years, many of which involve penetrating trauma. Education surrounding response to an MCI is broad and has many complex and ever-changing aspects that require staff to be updated on the most current information.

**Educational Goals:**

This curriculum is intended to maintain a knowledge base of MCI processes to mitigate degradation of necessary knowledge between hands-on MCI training.

**Educational Methods:**

The educational strategies used in this curriculum include short weekly refresher emails and optional external links for further reading.

**Research Methods:**

This content was evaluated for efficacy by administering electronic knowledge tests at baseline, mid-way (at 16 weeks), and at the end of the curriculum (32 weeks) via email. Additionally, brief content questions were asked in person while staff members were on shift throughout the entire study period, and a post-study survey was administered in order to obtain staff opinions on email length and training processes in general.

**Results:**

Scores for the knowledge tests were slightly higher at the end of the 32 weeks compared to baseline. Subjective feedback was positive overall at the end of the testing period.

**Discussion:**

Training and maintaining knowledge of roles and concepts of mass casualty incidents is vital since such events will never happen when expected. Short refresher emails allow educators to use spaced repetition and interleaving methods which have been shown to be a helpful adjunct to maintain knowledge, skills, and attitudes learned in more formal training.^1^,^2^

**Topics:**

Mass casualty incident, emergency department, decontamination, blast injury, media relations, biological agents, reprocessing, crisis standard of care, SALT (Sort, Assess, Lifesaving Interventions, Treatment/Transport) triage, personal protective equipment, disaster carts, airways, passive security, family reunification.

## USER GUIDE

**Table t1-jetem-5-3-c1:** 

List of Resources:	
Abstract	1
User Guide	3
Appendix A: Why You Matter	12
Appendix B: Getting to the Hospital	15
Appendix C: Job Action Cards	17
Appendix D: Decontamination	20
Appendix E: Self-Care	21
Appendix F: Personal Preparedness	24
Appendix G: Patient Identification	26
Appendix H: Child Management	27
Appendix I: Media Relations	29
Appendix J: Other Agencies	31
Appendix K: Personal Protective Equipment	32
Appendix L: Purging the ED	34
Appendix M: Disaster Carts	36
Appendix N: Call Out	40
Appendix O: Discharging	41
Appendix P: Operating Room (OR) Prioritization	43
Appendix Q: Passive Security	45
Appendix R: Evidence Preservation	46
Appendix S: Family Reunification	47
Appendix T: ^*^Coordinator Supplies	48
Appendix U: ^*^Answering Phones	50
Appendix V: ^†^Patient Belongings	51
Appendix W: ^†^Reprocessing	52
Appendix X: ^†^Trash	53
Appendix Y: ^‡^Biological Agents	54
Appendix Z: ^‡^Airways	56
Appendix AA: ^‡^Bleeding Control	58
Appendix AB: ^‡^Temporizing for the OR	61
Appendix AC: ^‡^Fluids	63
Appendix AD: ^§^Crisis Standard of Care	64
Appendix AE: ^§^Blast Injury	67
Appendix AF: ^§^SALT Triage	71
Appendix AG: ^§^Scope of Practice	74
Appendix AH: Incident Command	75
Appendix AI: Email Schedule	76
Appendix AJ: Post-Study Survey Results	78

[Table t1-jetem-5-3-c1]

**Learner Audience:**

- **Practitioners:** personnel who diagnose, prescribe and determine treatment plans. This includes but is not limited to emergency physicians, emergency medicine residents, physician assistants, and nurse practitioners.
- **Nurses:** This group also includes paramedics employed by and practicing in the emergency department.
- **Emergency room technicians (ERTs):** May also be known as nursing assistants or other similar job titles.
- **Coordinators:** personnel who perform the majority of clerical and communication duties, but do not perform direct patient care. May also be known as unit clerks, health unit coordinators or similar job titles.
- *Topics not intended for all disciplines are noted with the following:*
^*^Coordinators only
- ^†^ERTs only
- ^‡^Hands-on care personnel only (practitioners, nurses, and ERTs)
- ^§^Nurses and practitioners only

**Length of Curriculum:**

This curriculum is intended for use as weekly refresher emails spanning up to a 30-week period.

**Topics:**

Mass casualty incident, emergency department, decontamination, blast injury, media relations, biological agents, reprocessing, crisis standard of care, SALT (Sort, Assess, Lifesaving Interventions, Treatment/Transport) triage, personal protective equipment, disaster carts, airways, passive security, family reunification.

**Objectives:**

Appendix A: Why You Matter

1. Understand the importance of their job and response during an MCI
2. Describe where to find information on their role during an MCI

Appendix B: Getting to the Hospital

1. Anticipate challenges in getting to the hospital while responding for a mass casualty incident
2. Appreciate security challenges inherent to mass casualty incidents

Appendix C: Job Action Cards

1. Understand the purpose of the Job Action Card
2. Know where to locate Job Action Cards
3. Become familiar with the basic outline of a Job Action Card

Appendix D: Decontamination

1. Understand the "mantra" of decontamination
2. Identify the primary mission of decontamination
3. Identify two possible mistakes during a decontamination situation

Appendix E: Self-care

1. Understand the importance of good self-care practices, both in our everyday lives as well as within each stage of crisis within a disaster
2. Recognize the signs of burnout and secondary traumatic stress

Appendix F: Personal Preparedness

1. Describe the eight essential components of every disaster preparedness plan
2. Develop a personal emergency preparedness plan and kit

Appendix G: Patient Identification

1. Understand their role in identifying patients during an MCI
2. Recognize when to assign Doe names and when to use the patient’s ID (coordinators)
3. Identify and understand the correct process for arriving patients in the EMR (electronic medical record)

Appendix H: Child Management

1. Identify who takes care of unaccompanied pediatric patients in the ED
2. Identify where to send admitted and discharged pediatric patients

Appendix I: Media Relations

1. Understand the appropriate way for hospital employees to interact with social media, television and print media after or during a mass casualty incident

Appendix J: Other Agencies

1. Recognize the importance of collaborating and coordinating with other agencies in the face of disaster

Appendix K: Personal Protective Equipment

1. Recognize the importance of wearing proper personal protective equipment
2. Identify common and uncommon personal protective equipment and their uses during an MCI

Appendix L: Purging the ED

1. Recognize when to move stable patients to different areas of the ED
2. Understand when to discharge patients and how to do this efficiently
3. Understand how and when to admit patients to the floor and OR

Appendix M: Disaster Carts

1. Identify the basic types of items kept in disaster carts
2. Recognize a disaster cart

Appendix N: Call Out

1. Understand the importance of waiting for a call before going to the hospital
2. Understand the standard call out procedure

Appendix O: Discharging

1. Safely discharge patients both already in the ED and from the MCI
2. Recognize where to physically relocate discharged adults and children
3. Understand general discharge instructions and return precautions for common injuries from MCIs

Appendix P: Operating Room (OR) Prioritization

1. Consider who will make decisions about what patient goes to the OR and in what order in the case of an MCI
2. Prioritize injuries needing timely OR intervention in penetrating trauma - abdominal/junctional injuries, then chest injuries, then orthopedic/head injuries

Appendix Q: Passive Security

1. Describe the differences between active and passive security, and when each is utilized
2. Engage as an active member of our workplace’s security force

Appendix R: Evidence Preservation

1. Understand that all patient belongings need to be treated as evidence
2. Recognize that patient care comes first before adherence to evidence preservation guidelines
3. Identify what would be considered evidence and who makes that determination
4. Identify process for collection of evidence

Appendix S: Family Reunification

1. Establish a plan for how to reunite patients and their families
2. Consider logistical challenges that may be encountered
3. Prepare documents, checklists and directional signs to have in case of an MCI
4. Establish a communication plan for staff and media

Appendix T: ^*^Coordinator Supplies

1. Determine if necessary supplies are available
2. Understand how to use these supplies during an MCI
3. Identify 3 supplies that will be needed in an MCI

Appendix U: ^*^Answering Phones

1. Determine the appropriate response to phone calls inquiring about missing family or loved ones
2. Identify scripting to answer calls quickly & efficiently

Appendix V: ^†^Patient Belongings

1. Understand how to handle patient belongings
2. Understand procedures surrounding weapons or dangerous items found in patient belongings

Appendix W: ^†^Reprocessing

1. Recognize when to deviate from standard reprocessing procedures
2. Understand the importance of monitoring equipment supplies and anticipate needs

Appendix X: ^†^Trash

1. Distinguish the differences between the three possible types of trash that can accumulate during an MCI
2. Understand how to manage decontamination area trash, biohazard trash, and normal trash during an MCI

Appendix Y: ^‡^Biological Agents

1. Distinguish biological attacks from other mass casualty incidents
2. Identify signs of biological agents

Appendix Z: ^‡^Airways

1. Determine when patient’s airway establishment should be prioritized during an MCI
2. Review the basic logistics of airway management (ie, tools, medication, ventilators, etc)

Appendix AA: ^‡^Bleeding Control

1. Identify the tools available to assist with hemorrhage control
2. Understand the basic approach to controlling hemorrhage

Appendix AB: ^‡^Temporizing for the OR

1. Understand how to replace volume loss with fluids and, more importantly, blood
2. Identify ways to stop blood loss such as direct pressure, tourniquets, pelvic binders and interventional radiology procedures
3. Understand the importance of other interventions to buy time while waiting for the OR such as washouts, antibiotics, and pain medications

Appendix AC: ^‡^Fluids

1. Identify alternative methods to rapidly infuse fluids safely
2. Identify three ways to rapidly infuse fluids
3. Identify three potential issues to troubleshoot when fluids are not infusing

Appendix AD: ^§^Crisis Standard of Care

1. Explain why crisis standard of care is important
2. Identify basic differences between day-to-day care and standards of care in a crisis situation
3. Feel more comfortable abandoning usual practices in order to be efficient and care for the most patients possible

Appendix AE: ^§^Blast Injury

1. Recognize the wide variety of injuries that can present as a result of a blast injury or explosion.

Appendix AF: ^§^SALT Triage

1. Understand the process for triaging patients during an MCI
2. Identify the four main triage categories

Appendix AG: ^§^Scope of Practice

1. Understand the need for flexibility between roles during an MCI
2. Understand the golden rule of practice in MCI events
3. Understand MCI Chain of Command and who would make the decision to alter scope of practice

Appendix AH: Incident Command

1. Understand the importance of creating an emergency response structure

### Brief introduction

There have been an increasing number of mass casualty incidents (MCIs) occurring throughout the country in recent years, many of which involve penetrating trauma.^1^ Education surrounding response to an MCI is broad and has many complex and ever-changing aspects that require staff to be updated on the most current information. Large departments, such as the emergency department, struggle to make sure the right employees have the right information at the right time.^1^ Personnel of multiple disciplines (physicians, residents, nurses, coordinators and technicians) need to know a massive amount of information at any given time. Additionally, staff members work different shifts and need to have access to the information on a 24-hour basis. To manage this influx of information, many departments rely on email to communicate. Email is often used as a way to transfer knowledge to a large group of people; however, there are known limitations to this as many people will admit that they do not like getting excessive numbers of emails.^3^,^4^ The included content covers how our emergency department (ED) manages the dissemination of mass casualty incident planning information, and is meant to be adapted for use at other institutions.

### Problem identification, general and targeted needs assessment

Our team used the Kern framework to develop a curriculum for teaching our staff aspects of our Emergency Department’s Mass Casualty Incident plan.^5^ In the past, our department had emailed people our MCI policy, a 53-page document, as a supplement to the two MCI drills done annually, specialized annual training on radiation and chemical decontamination, and off-site training to personnel on our MCI and decontamination committee. Additionally, only managers are required to complete Federal Emergency Management Agency (FEMA) National Incident Management System (NIMS) training, which is not specialized for front-line patient care staff. The authors felt this was insufficient because this was too much information at once that was unlikely to be committed to memory. We also acknowledged that while a centralized document makes sense in theory, in an emergency ED staff don’t have the luxury of time to look things up and find the necessary information in a large document. In response, our MCI committee opted for a new approach to disseminate information about our disaster plans. We broke up this massive amount of information and policies into smaller, more digestible pieces.

This project was initially implemented as an Institutional Review Board-approved education research project assessing impact of length of email on information retention. We recognize that some people skim or do not read important emails, so these were written with ease of readability in mind by using an informal voice. Our hypothesis was that shorter emails would lead to a higher likelihood of being opened and read entirely by staff, leading to higher scores on the knowledge tests. After the study, all emails were available to staff on a department intranet site for reference.

A targeted needs assessment was completed prior to curriculum development. This needs assessment consisted of several steps. Initially, the lead author (JN) interviewed several staff members and administrators involved in the disaster planning process of the Emergency Department and hospital, ED MCI committee members from many disciplines, and frontline staff members in the emergency department. We reviewed after action reports from our hospital’s yearly MCI drills to identify performance gaps. Finally, we wrote a contest test that served as the pre-test for the education research projects. Test questions were written by a multidisciplinary team and tested on members of the disaster committee for accuracy prior to dissemination to the staff. Front-line staff members were offered the opportunity to voluntarily participate in the written pre-test. Curriculum content was then finalized and reviewed by MCI committee staff prior to dissemination.

### Goals of the curriculum

The global purpose of this curriculum is to maintain emergency department staff knowledge on key concepts involved in response to mass casualty incidents between hands-on training drills.

### Objectives of the curriculum

Appendix A: Why You Matter

1. Understand the importance of their job and response during an MCI
2. Describe where to find information on their role during an MCI

Appendix B: Getting to the Hospital

1. Anticipate challenges in getting to the hospital while responding for a mass casualty incident
2. Appreciate security challenges inherent to mass casualty incidents

Appendix C: Job Action Cards

1. Understand the purpose of the Job Action Card
2. Know where to locate Job Action Cards
3. Become familiar with the basic outline of a Job Action Card

Appendix D: Decontamination

1. Understand the "mantra" of decontamination
2. Identify the primary mission of decontamination
3. Identify two possible mistakes during a decontamination situation

Appendix E: Self-Care

1. Understand the importance of good self-care practices, both in our everyday lives as well as within each stage of crisis within a disaster
2. Recognize the signs of burnout and secondary traumatic stress

Appendix F: Personal Preparedness

1. Describe the eight essential components of every disaster preparedness plan
2. Develop a personal emergency preparedness plan and kit

Appendix G: Patient Identification

1. Understand their role in identifying patients during an MCI
2. Recognize when to assign Doe names and when to use the patient’s ID (coordinators)
3. Identify and understand the correct process for arriving patients in the EMR (electronic medical record)

Appendix H: Child Management

1. Identify who takes care of unaccompanied pediatric patients in the ED
2. Identify where to send admitted and discharged pediatric patients

Appendix I: Media Relations

1. Understand the appropriate way for hospital employees to interact with social media, television and print media after or during a mass casualty incident

Appendix J: Other Agencies

1. Recognize the importance of collaborating and coordinating with other agencies in the face of disaster

Appendix K: Personal Protective Equipment

1. Recognize the importance of wearing proper personal protective equipment
2. Identify common and uncommon personal protective equipment and their uses during an MCI

Appendix L: Purging the ED

1. Recognize when to move stable patients to different areas of the ED
2. Understand when to discharge patients and how to do this efficiently
3. Understand how and when to admit patients to the floor and OR

Appendix M: Disaster Carts

1. Identify the basic types of items kept in disaster carts
2. Recognize a disaster cart

Appendix N: Call Out

1. Understand the importance of waiting for a call before going to the hospital
2. Understand the standard call out procedure

Appendix O: Discharging

1. Safely discharge patients both already in the ED and from the MCI
2. Recognize where to physically relocate discharged adults and children
3. Understand general discharge instructions and return precautions for common injuries from MCIs

Appendix P: Operating Room (OR) Prioritization

1. Consider who will make decisions about what patient goes to the OR and in what order in the case of an MCI
2. Prioritize injuries needing timely OR intervention in penetrating trauma - abdominal/junctional injuries, then chest injuries, then orthopedic/head injuries

Appendix Q: Passive Security

1. Describe the differences between active and passive security, and when each is utilized
2. Engage as an active member of our workplace’s security force

Appendix R: Evidence Preservation

1. Understand that all patient belongings need to be treated as evidence
2. Recognize that patient care comes first before adherence to evidence preservation guidelines
3. Identify what would be considered evidence and who makes that determination
4. Identify process for collection of evidence

Appendix S: Family Reunification

1. Establish a plan for how to reunite patients and their families
2. Consider logistical challenges that may be encountered
3. Prepare documents, checklists and directional signs to have in case of an MCI
4. Establish a communication plan for staff and media

Appendix T: ^*^Coordinator Supplies

1. Determine if necessary supplies are available
2. Understand how to use these supplies during an MCI
3. Identify 3 supplies that will be needed in an MCI

Appendix U: ^*^Answering Phones

1. Determine the appropriate response to phone calls inquiring about missing family or loved ones
2. Identify scripting to answer calls quickly & efficiently

Appendix V: ^†^Patient Belongings

1. Understand how to handle patient belongings
2. Understand procedures surrounding weapons or dangerous items found in patient belongings

Appendix W: ^†^Reprocessing

1. Recognize when to deviate from standard reprocessing procedures
2. Understand the importance of monitoring equipment supplies and anticipate needs

Appendix X: ^†^Trash

1. Distinguish the differences between the three possible types of trash that can accumulate during an MCI
2. Understand how to manage decontamination area trash, biohazard trash, and normal trash during an MCI

Appendix Y: ^‡^Biological Agents

1. Distinguish biological attacks from other mass casualty incidents
2. Identify signs of biological agents

Appendix Z: ^‡^Airways

1. Determine when patient’s airway establishment should be prioritized during an MCI
2. Review the basic logistics of airway management (ie, tools, medication, ventilators, etc)

Appendix AA: ^‡^Bleeding Control

1. Identify the tools available to assist with hemorrhage control
2. Understand the basic approach to controlling hemorrhage

Appendix AB: ^‡^Temporizing for the OR

1. Understand how to replace volume loss with fluids and, more importantly, blood
2. Identify ways to stop blood loss such as direct pressure, tourniquets, pelvic binders and interventional radiology procedures
3. Understand the importance of other interventions to buy time while waiting for the OR such as washouts, antibiotics, and pain medications

Appendix AC: ^‡^Fluids

1. Identify alternative methods to rapidly infuse fluids safely
2. Identify three ways to rapidly infuse fluids
3. Identify three potential issues to troubleshoot when fluids are not infusing

Appendix AD: ^§^Crisis Standard of Care

1. Explain why crisis standard of care is important
2. Identify basic differences between day-to-day care and standards of care in a crisis situation
3. Feel more comfortable abandoning usual practices in order to be efficient and care for the most patients possible

Appendix AE: ^§^Blast Injury

1. Recognize the wide variety of injuries that can present as a result of a blast injury or explosion.

Appendix AF: ^§^SALT Triage

1. Understand the process for triaging patients during an MCI
2. Identify the four main triage categories

Appendix AG: ^§^Scope of Practice

1. Understand the need for flexibility between roles during an MCI
2. Understand the golden rule of practice in MCI events
3. Understand MCI Chain of Command and who would make the decision to alter scope of practice

Appendix AH: Incident Command

1. Understand the importance of creating an emergency response structure

### Educational strategies

Please refer to the linked objectives and educational strategies.

### Results and tips for successful implementation

We sent weekly emails to all patient care staff in the Emergency Department for 32 weeks. These emails were sent from a dedicated email address within our institution’s firewall. To attempt to measure how often and when emails were read, we used a commercial software for email marketing (MailChimp, Atlanta, GA). This attempt was unsuccessful because several emails were short enough to be completely read in the preview pane of our email client, causing most readers to never fully open the email and scroll to the bottom, which would have triggered the marketing software to mark an email as read. Readership was also impacted by our choice to use a project-specific email address. Our information technology and services department launched an extensive campaign to improve staff member ability to recognize and not open suspicious emails to minimize “phishing” attacks. Our email curriculum was from an unfamiliar email address and routed through marketing software. After several weeks of emails with readership rates, we discovered that many ED staff thought our emails were suspicious and therefore intentionally did not open them. We highly recommend potential users of this email curriculum partner with the information technology professionals within their facility to maximize the ability for hospital staff to recognize these emails as legitimate, relevant, high-yield, and worth opening.

We attempted to evaluate the effectiveness of this curriculum on several levels.^6^ Subjective feedback was obtained with ad hoc discussions with staff members in meetings and an electronic survey at the end of the curriculum. We also attempted to evaluate knowledge retention by staff members as well as change in behavior of learners. We did electronic tests before, midway, and after the email curriculum. However, these tests were voluntary. Also, as the email curriculum was occurring, staff members were highlighting gaps and areas for improvements, which led to substantive improvements to our MCI plans, which incidentally changed several answers to questions that were used in the pre-test. This led to us not being able to fairly compare test scores pre- and post-curriculum.

Our final attempt to assess behavior change was to compare findings on the After Action Reports from our annual large-scale MCI drills before and after the email curriculum. The drill that was to be held immediately after the email curriculum was unfortunately postponed due to severe weather, leading to a significantly altered type of drill which made direct comparisons impossible.

The text in the appendices is based on the emails sent to our staff, but has been adapted for general use at any institution. We found keeping emails brief while including essential information in an informal voice is ideal in settings where employees have limited time or energy to read many emails on policy. Users of this curriculum need to determine if this is appropriate for their institution and adapt the text as necessary. Users need to closely review and alter the content to fit their department prior to use. Any terms in [brackets] should be replaced with institution specific terminology and processes. Any other sections not applicable to specific institutions may be altered or removed as appropriate. This content is meant to supplement substantial hands-on training; therefore, we do not recommend this curriculum as the sole method of training for such events.

### Program Evaluation

Response to the voluntary perception survey was low at 12.3% (54/442). Sixty-two percent of respondents felt our Emergency Department was “more prepared than last year,” while 36% felt it was “equally prepared.” Shorter emails were preferred, with 66% preferring shorter emails and 32% having no preference. Only 2 % preferred the longer emails. More detailed survey results can be found in Appendix AJ.

### Associated Content

We included specific emails covering topics facing most emergency departments in the United States with specific details from our department redacted. We highly encourage customization to fit the specific needs of the user’s department. The questions to the knowledge tests were not included for sake of brevity, but these can be provided upon request. Many of these are organization specific, so we suggest the users write their own specific to their department or facility.

## DIDACTICS AND HANDS-ON CURRICULUM

### Appendix A Why You Matter

**Note: Information in this appendix may be institution specific. Please evaluate content relevance and insert institute specific information as needed**

#### Objectives

At the end of this activity, learners will be able to:

1. Understand the importance of their job and response during an MCI
2. Describe where to find information on their role during an MCI

Each and every member of our team in the ED is crucial for successful operations during day-to-day work, and this is especially true in the event of an MCI. No single role is more important than another and **everyone** has a part to play in making things run smoothly. We all must rely on each other to provide the best care possible to our patients. One of the goals of this project is to ensure that all members of our team know why they are important in an MCI event. According to a study, although 53% of health care respondents were willing to assist in an MCI, only 23% had the knowledge or confidence to respond^1^. If you know why you are important, you will have more confidence and be more likely to respond **when we need you most.**

General-expected by all roles

- Please be flexible - we may use you in a variety of different locations or roles.
- We might need your help right away or ask you to come later (to relieve staff on site).
- Communication with other roles and specialties will be key!
- If you don’t know what to do during an MCI, check your Job Action Card (JAC) first, then ask your lead.
  ○ Refer to Appendix C for more information on Job Action Cards.
- We need you in the ED! Unless specifically directed by ED leadership, do not leave the department. Other staff will transport patients.

[The following section can be tailored for the role the email is directed to]:

#### Coordinator Specific Roles

Coordinators are some of the most important members during an MCI because you help keep the huge influx of patients organized and accounted for. We will be counting on you to assist with **communication** across roles and specialties. *You are the eyes and ears of our department* and are the best people to have a handle on how the department is functioning as a whole. Some of your most important roles include:

- Registering all arriving patients in the electronic medical record (EMR) (use Doe name if needed)
- Placing a wrist band on all patients
- If the EMR is down, helping with paper documentation (printing patient labels, labeling nursing records and ensuring that these stay with the patient)
- Registering patients as a disaster victim or un-related to the disaster
- Updating Doe patients if identifying information is obtained
- Coordinating and communicating with other departments such as radiology, OR, security, etc.

#### ERT Specific Roles

During a mass casualty incident, *ERTs are the grease that keeps the wheels of the ED moving*. ERTs know where all of the equipment is or how to get it. As an ERT, you also are aware of when to get more supplies based on your experience during normal operation. Most importantly, you know where the disaster supplies are kept [list location within your department here]. *Without you, our department would grind to a halt during an MCI*! Some of your most important roles include:

- Check vital signs and place patients on monitors, if needed
- Collect supplies and deliver them to needed areas
- Keep track of patient belongings
  ○ This will help with investigations, protect patient’s identities and ensure that patients do not lose anything.
  ○ Grouping a patient’s belongings keeps the ED cleaner and easier to maneuver within
- Assist with quick turnover of rooms – immediately remove them from the EMR once they have physically left the room
- Be the extra set of hands and eyes to help everyone else fulfill their role!

#### Practitioner Specific Roles

While our role during an MCI is *similar to our day-to-day roles as practitioners*, the rest of the ED staff will likely look to us for direction in this chaotic environment. As such, practitioners will be expected to act as **leaders** during the MCI. It is up to you to *direct the care of your patients* and make critical decisions in their treatment. You will be expected to provide care for the massive influx of patients that we may receive. You may serve as the lead attending physician until ED leadership arrives or work in triage to complete medical screening exams for patients who will be referred to alternative care facilities. Some of your most important roles include:

- Developing care plans for patients in the department
- Admitting or discharging patients as able (refer to Appendix L for more information about purging the ED)
- Considering the possibility of chemical/biological/radioactive exposures and need for decontamination
- Managing scarce resources and using crisis standards of care to make decisions in line with these guidelines (refer to Appendix AD for more information about crisis standard of care)
- Communicating with charge RN, PFC (patient flow coordinator), ambulance dispatch, and trauma teams
- Helping coordinate patient care teams
- *Residents and PAs*- you will function in your normal patient care roles, unless directed otherwise
  ○ Call all off-service ED residents in the hospital, with the exception of the Surgical Intensive Care Unit (SICU) resident, to report to the ED.
- *Medical students* should not be assigned as primary practitioners, but can perform roles such as suture technicians or other assistant roles, as directed by resident or staff.

Be a leader! Inspire your team. We kick off this email campaign with this topic very intentionally. Studies have shown that staff members are more likely to respond to a disaster if they believe their job is important. While most of the patient care will be delivered by nurses and providers, do not forget the ERTs, coordinators and everyone else who makes our ED run. You will turn to those familiar faces that know where something is and how to get something done. *Make sure they know just how much you need them*.

#### RN Specific Roles

Nurses are the largest group of health care workers and you are at the forefront of medical care, playing a key role in major disaster relief operations^2^. You provide the *majority of the hands-on care to our patients* during an MCI. During an MCI, you can expect your workload to increase. Some of your most important roles include:

- Starting triage to organize the influx of patients (refer to Appendix AF for more information on SALT triage)
- Providing both physical and mental health care
  ○ Triage psychologically wounded to a behavioral health treatment site (if available)
- Using RN initiated (RNI) orders as appropriate
- Managing scarce resources and considering MCI standards of care (refer to Appendix AD for more information about crisis standard of care)
- Considering the possibility of chemical/biological/radioactive exposures and need for decontamination, assisting with decontamination
- Communicating with PFC, ambulance discharge and OR staff
- Charting identifying information about patients, as able, to assist with family reunification (refer to Appendix S for more information on family reunification)

#### Paramedic Specific Roles

Paramedic roles are intentionally less defined in an MCI. We understand that many of you have other jobs in EMS. During an MCI, you probably need to be with your EMS service. *Those of you who can respond here*, *please do!* With your skill set, you are the ultimate utility players. We aren’t defining your role here because we don’t know where to start. You can do so many things. There will be plenty of work to do.

#### Conclusion (for all)

If you remember nothing else, please remember that *you are important and we need you* during an MCI! As Emergency Department staff, you are the best equipped of anyone in the hospital to help care for these patients. Each of you has a special skill set and can help care for a large number of patients in the event of a disaster!

### Appendix B Getting to the Hospital

**Note: Information in this appendix may be institution specific. Please evaluate content relevance and insert institute specific information as needed**

#### Objectives

At the end of this activity, the learner will be able to:

1. Anticipate challenges in getting to the hospital while responding for a mass casualty incident
2. Appreciate security challenges inherent to mass casualty incidents

Based on data from actual mass casualty incidents, the first wave of casualties (those requiring only minor care) may arrive at the hospital within the first 15–30 min. Approximately 50% of acute casualties may arrive at medical facilities within 60 min, 50%–80% within 90 min, and most arrive within 1–4 hours.^1^ Therefore, in a mass casualty incident, the **majority of patients will receive their initial life-saving care by the people working in the ED at the time of the incident.** However, your colleagues in the ED will still need your help!

#### Getting TO the hospital

When you receive the call, you will be asked for an estimate of when you can get here. We know you can’t teleport. The [committee or entity that makes disaster plans] plans around the fact that it will take you some time to get here. After all, you are expected to drop what you are doing, arrange care for any dependents, get dressed, arrive at the hospital and mentally prepare for what you are about to face.

Between ambulances, police, patients, loved ones, the press and security, getting to the hospital campus will be a nightmare. We cannot really specify what routes will be open or closed because that will vary by scenario.

Driving here? Parking will be hit or miss. Depending on the event, we cannot guarantee ramp gates will open. Ramps will fill up quickly. Roads to the parking ramps may be blocked. *Anticipate problems*. (You might just want to get a ride.)

#### Getting INTO the hospital

During a mass casualty incident alert, the hospital will have limited access for everyone’s safety. Ambulances will be able to drop off patients at the ambulance entrance. Your badges should work for entering the building. But for everyone else, [name of appropriate door] will serve as the only open entrance to the hospital. As people come through the doors, they will be directed along three (3) pathways.

1. Patients: [where patients will go]
2. Staff
  - ED staff: checked by security, then go to [location] for assignments
  - Hospital staff: [where non-ED staff should go]
  - Family members: directed or escorted by [who] to [location]
3. Press: [where members of the media should go]

#### To make it through security as quickly as possible

1. Badge in through your typical door. Don’t use the ambulance entrance please.
2. If your badge doesn’t work, go to the [main door]. [Insert diagram]. Do not call security to buzz you in; they will be really busy.
3. Show the staff your badge and tell them you work in the ED. Maybe security will recognize you, maybe they won’t. Don’t be offended. They are stressed and trying to keep us safe.
4. NO CONNIPTION FITS! This situation is already drama packed, so don’t add to it.
5. Once through, go to [location].

See you in the ED…eventually.

### Appendix C Job Action Cards

**Note: Information in this appendix may be institution specific. Please evaluate content relevance and insert institute specific information as needed**

#### Objectives

At the end of this activity, the learner will be able to:

1. Understand the purpose of the Job Action Card
2. Know where to locate Job Action Cards
3. Become familiar with the basic outline of a Job Action Card

**Keep Calm and Find Your Job Action Card**

#### Where do I go and what do I do?

For many, this might be the first reaction to an MCI. More nurses, techs, and practitioners will also be arriving to help with the surge. Effective organization of our personnel during this time is crucial to providing care. The primary source of information dictating an individual’s role during an MCI is the Job Action Card (JAC). The JAC is actually a full-size piece of paper with all the relevant information about where to go and what to do for all emergency department personnel. JACs can be found located in the disaster carts (refer to Appendix M for more information on disaster carts). JACs can be found on the [institution specific intranet, if available] and are updated often, so memorizing every detail ahead of time is not necessary. However, it is important to have looked at a JAC before scrambling to find yours during an MCI. Below is a sample of the JAC for the Charge Nurse.

[This includes an example of our Job Action Cards; please insert your own.][Fig f1-jetem-5-3-c1]

#### IMPORTANT NOTE

Many of the JACs are multiple pages and are written FRONT and BACK. Read the whole thing![Fig f2-jetem-5-3-c1]

Additionally, the disaster cart in [area] is the source for a complete packet of information for all of the supervisory positions. This packet includes the JAC, a complete copy of the MCI disaster policy, and any relevant forms for a particular job. All the supervisory positions come with an orange vest. Below is a picture of one of the containers within the disaster cart that keeps the JACs as well as position specific clipboards and vests.[Fig f3-jetem-5-3-c1]

### Appendix D Decontamination

**Note: Information in this appendix may be institution specific. Please evaluate content relevance and insert institute specific information as needed**

#### Objectives

At the end of this activity, the learner will be able to:

1. Understand the "mantra" of decontamination
2. Identify the primary mission of decontamination
3. Identify two possible mistakes during a decontamination situation

#### Decontamination Refresher

Patients potentially exposed to chemical, radiological, nuclear and/or biological agents need to be handled very specifically to protect the patient and anyone around him/her.

**Remember the mantra of decontamination: “I DON’T KNOW.”**

- *I don’t know what the exposure actually is*: Most patients will not come in with safety data sheets (SDS). Even if we know what the chemical is, it takes time to find the right information.
- *I don’t know if this is dangerous or not*: It takes time to figure this out too. If we aren’t sure, decontaminate the patient anyway to avoid continued exposure to staff.
- *I don’t know all the specifics of decontamination*: Many staff members have been given extra training in decontamination. You can too! Contact your [appropriate person] for more information.

We need to care for the patient but our **primary** mission is to protect our staff and protect the house. **Decontamination trumps patient care. PERIOD.** Contaminated areas of the ED need to be put out of commission until cleared. Think about traffic patterns in the ED. If contaminated patients/items circulate through the ED, downstream events can be terrible. You are exposed, your coworkers become exposed, you go home and expose family. Once discovered, everywhere that the patient was needs to be shut down. When decontamination happens, the decontamination team lead is in charge of decontamination procedures. The decision about doing decontamination is either right or wrong. If we choose correctly, good for us. However, very often we will be wrong. If we are wrong, we can be wrong in two ways. Either we fail to decontaminate a patient who needs it (under-call) or we decontaminate a patient who did not need it (over-call.) With an under-call, we contaminate ourselves, our coworkers and our environment. If we over-call, we have a wet patient. When in doubt, decontaminate.

### Appendix E Self-Care

**Note: Information in this appendix may be institution specific. Please evaluate content relevance and insert institute specific information as needed**

#### Objectives

At the end of this activity, the learner will be able to:

1. Understand the importance of good self-care practices, both in our everyday lives as well as within each stage of crisis within a disaster
2. Recognize the signs of burnout and secondary traumatic stress

#### Making Self-Care a Priority

In our chosen vocation, we have the rewarding privilege to care for others and help them in their most vulnerable states. However, it is no surprise to each of us that with this comes the burdens of emotional exhaustion and significant burnout. As emergency responders and healthcare professionals, we must make self-care in our daily lives, at work and at home, a priority now so that we may prepare ourselves as best as possible should we be faced with a more traumatic event such as an MCI. We experience terrible things in the ED. In a Mass Casualty Incident, those things are magnified in both quantity and quality.

**Picture it: Severely injured children and adults, dead bodies or body parts, loss of colleagues, unknown fate of loved ones.**

*This will mess with us. And in some cases, it likely already has.*

As a team, we must assume responsibility for our own self-care. It is essential that we, as individuals and co-workers, utilize the following simple methods to recognize, monitor, and maintain health prior, during, and after such an experience.

#### Before the Crisis

- Develop preparedness plan and kit
- Take advantage of any pre-disaster training
- **Be aware** of your own emotional reactions and triggers
- **Connect with others**
- **Maintain a balance** between your professional and personal lives

#### During the Crisis

It is normal for responders to experience stress during a crisis. Recognize the signs of burnout and secondary traumatic stress!^1^ We can manage stress by taking breaks and watching out for one another by limiting the amount of time we work alone, and instead, work in teams. We can pace ourselves between low and high-stress activities. Try to stay in contact with family and friends on your breaks--it may put your mind at ease if you know they are safe.

How to recognize the signs of…

Burnout:

- Sadness, depression, apathy
- Easily frustrated or irritable
- Isolation or disconnection from others
- Tired, Exhausted, or Overwhelmed
- Feeling: you are a failure, helpless, inadequate
- Needing substances such as alcohol or drugs to cope

Secondary Traumatic Stress:

- Excessive worry or fear
- Easily startled or “on guard”
- Physical signs of stress
- Nightmares, flashbacks, recurrent thoughts about traumatic event
- Feeling that others’ trauma is your own

**It is extremely important to remind yourself:**

- It is not selfish to take breaks--working all the time does not mean you will make your best contribution. Other members of the team are also helping in the response.
- Survivors are not more important than your own needs and well-being.
- It is okay to draw boundaries and say “no.”

Recognize and accept what you cannot change--the chain of command, structure, waiting, equipment failures, etc.

#### After the Crisis

Many people will experience *intrusive symptoms*, which can be physical, cognitive, emotional, or behavioral symptoms of stress. **These are *****normal***** responses to an *****abnormal***** situation.** Give yourself time to debrief and reflect on how the event changed you. Take time off or away from work to allow you the time to regroup, to recharge, and to heal from this experience.

### Appendix F Personal Preparedness

**Note: Information in this appendix may be institution specific. Please evaluate content relevance and insert institute specific information as needed**

#### Objectives

At the end of this activity, the learner will be able to:

1. Describe the eight essential components of every disaster preparedness plan
2. Develop a personal emergency preparedness plan and kit

#### Ensuring Personal Preparedness during a Mass Casualty Incident

Within a few seconds and often without warning, disaster can strike. Each year, hundreds of thousands of lives are disrupted due to the effects of these emergency situations. 1 By ensuring our own personal preparedness, we can lessen the impact on our family, on our workplace, and on our community.

**Picture it: Mass bombings in the [local area]. Multiple fatalities and mass injuries. You are needed in the ED immediately.**

How do you feel right now? Do you have children, pets, and/or elder family members for whom you provide care? Do you feel like you are prepared enough to leave your loved ones during this emergency event and help our community in your role as a health professional?

**Solution:** Let’s create a simple, yet comprehensive plan to maximize your plan for personal preparedness should a real disaster situation occur. Here are a few guidelines and resources to get you started:

There are eight essentials of your preparedness plan: 1, 2

1. 3 Days of Food (for each member of your family)
2. 3 Days of Water (for each member of your family)
3. Flashlight
4. Radio
5. First Aid Kit
6. Emergency Childcare Plan
7. Emergency Pet Care Plan
8. Emergency Elder Care Plan

Designing a plan can be overwhelming and time-consuming, and we all can appreciate how busy you are in your life! That is why the Red Cross has developed a tool to aid you and your family in creating the best disaster kit. You can use this tool to prepare, in a series of small steps, over the next 21 weeks.^2^

The Office of Emergency Preparedness at University of California-Los Angeles Health System designed a program titled “Get Ready, Stay Ready” to help you with your personal preparedness efforts by providing a *comprehensive 12-month guide*.^1^ Each month, you can take one step to putting together an ***all-hazards*** emergency plan and kit that will prepare you for the many different disaster scenarios.

And lastly, trying to locate important documents can be difficult and tedious enough outside of a disaster; thus, protecting these documents as part of your emergency preparedness plan is essential. Examples of these documents may include legal records, insurance policies, property records, medical information, financial records, and many more^3^. To assist you with this process, the Department of Homeland Security’s Federal Emergency Management Agency (FEMA) has developed a family emergency plan to keep vital records organized.^4^

We hope that we are never faced with the tragic situation above, but if we are, our goal is to be as prepared as possible. Through these preparedness efforts, we can help reduce the fear and anxieties these unknown conditions raise, as well as reduce the immediate and long-term risks that threaten you and your family. 1 You have entered a rewarding vocation that is dedicated to its community during times of great need, and for that service, you will always find great honor and appreciation.

### Appendix G Patient Identification

**Note: Information in this appendix may be institution specific. Please evaluate content relevance and insert institute specific information as needed**

#### Objectives

At the end of this activity, the learners will be able to:

1. Understand their role in identifying patients during an MCI
2. Recognize when to assign Doe names and when to use the patient’s ID (coordinators)
3. Identify and understand the correct process for arriving patients in the [EMR]

All patients will be “arrived” into the [EMR] prior to entering treatment areas. This process will be followed for both triage arrivals and ambulance arrivals. Doing this allows other people throughout the hospital to see how many patients are arriving. This also will hopefully minimize how many phone calls to the ED asking about patient volume because this information can be seen in the [EMR]. Most patients will be given “Doe names” unless ID is present with them. IDs used will include a *recognizable* picture ID, family present and able to identify patient with name and DOB, or if the patient is able to speak for themselves and provide name and DOB. If the information only matches one person, use it. If there are multiple matches, create a new chart. With the current process, all red-tagged patients will automatically be given Doe names to prevent delays with care. Wristbands will be placed on all patients.

With initial patient arrival, speed is key. Only coordinators will be arriving patients so other staff can focus on triage efforts.

It is possible that severely injured patients may deteriorate and need to get a gray or black tag, meaning they will likely die. This is not the fault of any staff member, including the coordinator. **If the patient is not stable enough to make it through the expedited triage process, they are not, and were not, going to survive.**

Eventually, the hospital, law enforcement, or the Medical Examiner will need to identify patients. We can make this easier and more streamlined if we keep any belongings with patients throughout their course in the ED. Do not take any photo IDs away from patients, even to make copies. If you find any identifying information, alert the coordinator in your pod.

### Appendix H Child Management

**Note: Information in this appendix may be institution specific. Please evaluate content relevance and insert institute specific information as needed**

#### Objectives

At the end of this activity, the learner will be able to:

1. Identify who takes care of unaccompanied pediatric patients in the ED
2. Identify where to send admitted and discharged pediatric patients

The phrase “children are not tiny adults” applies to mass casualty situations. Children do not respond to emergencies the same way adults do; they don’t understand what is going on, don’t follow commands well, may be scared or try to hide from people trying to help them. Sounds like just the kind of additional chaos you want in the ED during an MCI event, right? Depending on the situation, however, we may be faced with multiple pediatric patients or stranded children, so we need a plan for how to care for them. *Anticipate that we will have some pediatric patients!*

#### Dealing with Pediatric Patients in the ED

- Pediatric consent to treat is assumed (efforts to obtain consent in the non-emergency patient will be made as time allows)
- Triage should send a pediatric supply container with each pediatric patient to ensure that appropriately sized equipment is available.
- One family member should be allowed to stay with each child
  ○ The family member must be identified by the patient, be able to show photo identification, or be able to identify the patient by clothing, jewelry, or body characteristics
- Remove face mask and head gear (if safe and appropriate) to ease fears. Masks can be scary to kids!
- Consider placing intraosseous (IO) lines instead of taking the time to place an IV
- Decontamination processes may need to be altered--lower pressure, warmer water or allow staff to stay in the decontamination space with children who cannot follow commands

[Discuss with your pediatric staff how/if they will be involved in MCIs. Consider if pediatric intensivists/pediatricians will stay in the ED or remain in the Pediatric Intensive Care Unit (PICU).]

As a quick refresher on **pediatric physiology**, children have: 1

- Larger heads making them more susceptible to head trauma
- More body surface area making them more prone to hypothermia
- Larger and less well protected internal organs
- Faster respiratory and heart rates – this may make them more prone to toxin exposure

#### Admitting Pediatric Patients

[Discuss with your pediatric staff where and to what service pediatric patients will be admitted (peds vs trauma). Have a contingency plan for overflow units and need for pediatric RNs.]

#### Discharging Pediatric Patients

Discharged unaccompanied pediatric patients should be moved to a Pediatric Safe Area (staffed by a hospital employee). [Establish where this area will be in your hospital.]

- This area should have play pens, toys, snacks, juice, formula, diapers, etc.
- Separate Job Action Cards should be available for employees staffing the Pediatric Safe Area (refer to Appendix C for more information on Job Action Cards).
- Before the minor can leave with an adult, the hospital employee will ensure that the adult is identified by the patient or shows photo identification and signs the patient out on the [Pediatric Safe Area Registry, or similar form] [Attach institution specific form].

Now we’re ready to provide the best care to our smallest victims!

### Appendix I Media Relations

**Note: Information in this appendix may be institution specific. Please evaluate content relevance and insert institute specific information as needed**

#### Objectives

At the end of this activity, the learner will be able to:

1. Understand the appropriate way for hospital employees to interact with social media, television and print media after or during a mass casualty incident

#### Media Inquiries Related to a Mass Casualty Incident

Media presence after a crisis is unavoidable. However, the treatment our hospital receives by the media and the resulting public opinion is within our control. All media inquiries should be relayed to the public relations office or incident command. The media will categorize anyone who works at our hospital as an official representative of the entire organization. The media will form the national narrative on this type of event. It is imperative to understand how important it is for our organization to speak with the media in a composed and structured fashion. This document will outline important aspects of dealing with the media during a mass casualty incident.

#### The Media

- At no time will the media be allowed through any patient care or treatment area.
- The media should be kept outside of the facility and they shall not interfere with the health and welfare of the general patient population.
- Media may attempt to gain access to the emergency department/patient care areas by pretending to be family members of patients.
- Any person seen videotaping/recording events in patient care areas will be asked to leave.
- The public relations office/hospital administration/incident command will handle all media inquiries.
- Employees should not speak with the media and should defer all solicited media inquiries to the proper channels.

#### Crisis Communication Team

- Crisis communication requires a plan and a team
- A crisis communication plan provides detailed procedures for communicating with employees, patients, family members and media after a crisis.
- The hospital public relations office in conjunction with the emergency department, hospital administration, and incident command will be in charge of ALL media communication.

#### Social Media

- Social media is an extremely powerful tool for disseminating information during a time of crisis.
- Many people receive a majority of their news from social media.
- Hospital employees should refrain from discussing any information related to a mass casualty incident on social media.
- All social media posts should be considered permanent public statements.
- If employees post any information they have about an MCI as a result of working at our hospital, they are effectively speaking on behalf of the entire organization.

### Appendix J Other Agencies

**Note: Information in this appendix may be institution specific. Please evaluate content relevance and insert institute specific information as needed**

#### Objectives

At the end of this activity, the learner will be able to:

1. Recognize the importance of collaborating and coordinating with other agencies in the face of disaster

Disaster can strike at any time. Multiple emergency response agencies from both the public and private sectors of multiple cities, including fire and police departments, emergency medical system, and volunteers, unite together to ensure the safety and health of the public. Effective coordination leads to effective response that saves lives.

#### Coordination in Disaster Planning and Response

Public safety agencies (fire/police departments, etc.) play vital roles in search and rescue, transport of casualties, decontamination, and providing emergency medical care and first aid. 1 Often there is involvement from the military, government agencies, or from local volunteers. Much of the policy and procedure guidelines that should be in place to ensure multiagency coordination and communication are often deficient. You may need to respond to a disaster someday and work with individuals from other agencies with whom you have never before worked. They may have different training methods, organizational structure, equipment, protocol, strengths, weaknesses, and may use different terminology and avenues of communication. 1 This can be complicated by the fact that most health care in the United States is provided by the private sector, one that is largely outside the direct operational and fiscal control of the government. 1 Therefore, we will need to be flexible and work within our Incident Command structure in the hospital to coordinate our efforts with those of the many agencies that are helping to respond to whatever we are facing.

### Appendix K Personal Protective Equipment

**Note: Information in this appendix may be institution specific. Please evaluate content relevance and insert institute specific information as needed**

#### Objectives

At the end of this activity, the learner will be able to:

1. Recognize the importance of wearing proper personal protective equipment
2. Identify common and uncommon personal protective equipment and their uses during an MCI

During an MCI, it is important to use the proper personal protective equipment (PPE). This is just a brief refresher list of common and uncommon PPE that you may use, and why you should use each individual piece. Remember to wash your hands or foam in/out before and after putting on any of this equipment. Isolation signs should still be used, and they tell you exactly what you need to wear before entering each room. It is vital that you read these and follow their requirements to prevent the spread of diseases among patients in our ED.

- **Gloves**: The most basic piece of PPE that should be used for all patient encounters. Always check your gloves for rips, tears, or punctures before beginning patient care. Change your gloves when they become significantly soiled with blood or other bodily fluids because this will help prevent accidental contamination of yourself or surroundings.
- **Gown:** The basic PPE that protects the wearer’s clothing and arms from contamination from an external source. These should be used for droplet, contact, enteric, and airborne isolation.
- **Basic Mask (w/face shield):** A basic mask should be used to cover your face to protect against droplets and larger particulates. These do NOT provide protection against airborne agents such as tuberculosis (TB). Masks with face shields are preferable for droplet precautions.
- **Shoe Covers & Hairnets:** Light covers for feet and hair to protect against contamination. These should be used in situations where you could be at risk for large exposure to bodily fluids such as trauma.
- **N95 Respirator**: An advanced mask that can filter airborne pathogens out of the air (TB, chickenpox/shingles, etc.) and requires fit testing. Any staff not fit tested or who failed fit testing must use a powered air-purifying respirator (PAPR) to enter an airborne precautions room.
- **Powered Air Purifying Respirator** (PAPR): A motorized, wearable air-filtering unit that can provide protection against a wide variety of airborne agents. Because there are many kinds of PAPRs, it is important to ensure that you are using the correct one. Some PAPRs are only designed to protect against airborne biological agents such as TB, while others can also protect against chemical and radiological agents.
- **Water Resistant Jumpsuit**: A white, fluid and tear resistant suit that functions as a full body gown. When wearing a jumpsuit, facility **provided boots or shoes** will be worn as well to protect your feet from harm and breaches in the suit. If also using a PAPR, these suits need to be donned before the PAPR. These suits should be used for decontamination procedures or full barrier precautions or if directed to do so by a supervisor.’

[Please insert institution specific pictures with brand names familiar to staff. Below are some examples][Fig f4-jetem-5-3-c1]

### Appendix L Purging the ED

**Note: Information in this appendix may be institution specific. Please evaluate content relevance and insert institute specific information as needed**

At the end of this activity, the learner will be able to:

1. Recognize when to move stable patients to different areas of the ED
2. Understand when to discharge patients and how to do this efficiently
3. Understand how and when to admit patients to the floor and OR

In the event of an MCI, we will need to optimize every bed in the ED to make as many as possible available for critically ill patients in need of acute care. This means clearing out current patients from the ED and creating an efficient flow plan for the influx of patients from the MCI. We will be making disposition decisions earlier on in the course of care than normal, which will also require cooperation from our colleagues in other specialties

Tips on how to clear out your [area of the ED] fast:

- Deviate from the standard of care (refer to Appendix AD for more information on crisis standard of care)
  ○ Chest pain, for example:
    ▪ Stable patient getting pulmonary embolism (PE) workup = consider subcutaneous anticoagulant injection and discharge
  ○ Closed fracture = splint and go (maybe after getting pieces in the correct time zone at least)
- Admits = go up now, no more workup
- Cab slips = Sure! Just leave
- [Insert other pertinent common barriers to discharge]

#### Admitting

The [PFC] and [person managing majority of admission, such as a triage hospitalist] will be the go-to people to assist with quick admissions. The goal is to get all pending admissions up to inpatient units ASAP. However, don’t expect your patients to magically vanish.

- Patients will be brought to the floor by float pool personnel (we don’t want any of our ED staff leaving the ED).
- We may need to cohort admits in a peripheral area of the ED while waiting for transporters.
- Full work ups may not be done at this point.
- Admit orders likely will not happen.
- Consider having a single point person from medicine service come to ED to get high-level sign out on all patients coming in. Typical telephone sign out will not be feasible.
- Keep in mind logistics- *someone* still has to order a bed, physically take the patient upstairs and everyone will be busy! This may take time.

#### Discharging

Get all stable patients out of the ED ASAP. During an MCI, it is appropriate to refer them to urgent care or primary care clinic. The idea is to give verbal (and possibly pre-written) discharge instructions as you are rapidly showing someone the door [Which door? Consider the logistics of this and that it might vary throughout the incident] (Refer to Appendix O for more information on discharging).

- Adult patients will be discharged and sent out to wait in a defined area of the hospital (no waiting for rides in the ED).
- Pediatric patients without an adult present will be discharged to a Pediatric Safe Area (refer to Appendix H for more information on child management)..
- Consider how your sort-of intoxicated patient will cope in the newly chaotic world outside. Our partners in public service won’t have time to deal with them. The right answer might be finding a quiet corner for them to safely continue to sober up.

#### Move to separate area of the ED

Depending on the MCI, we may not have time to purge and prepare. Another option is to divide and conquer. The lead MD and charge RN can pick one area to clear ASAP to receive initial victims.

- Move stable patients (who still need to be in the ED) to another area
  ○ Assign skeleton staffing to this area. Consider resident physicians or advanced practice providers with fewer years of experience.
- Depending on how many patients we are expected to get, you may consider keeping intubated or hemodynamically unstable patients in high acuity areas.
- Consider placing two patients per room
- Use chairs as appropriate
- Write a quick note if possible and share in chart since face to face sign outs likely won’t happen

In short, make disposition decisions earlier than normal and clear the department.

### Appendix M Disaster Carts

**Note: Information in this appendix may be institution specific. Please evaluate content relevance and insert institute specific information as needed**

#### Objectives

At the end of this activity, the learner will be able to:

1. Identify the basic types of items kept in disaster carts
2. Recognize a disaster cart

MCI’s will bring a large influx of trauma patients into the emergency room. The purpose of the disaster carts is to create a central depot of critical tools to help us do our jobs. In addition to medical equipment, job action cards and MCI policy documents [insert other major categories of supplies] are also contained in these disaster carts. We have [insert number of carts] disaster carts that are stocked and ready for deployment into the ED in the event of an MCI. They will be found in different strategic locations in the ED.

[Insert where disaster supplies can be found.]

When an MCI is called, [personnel] will bring them to the ED.

The [number of carts] disaster carts are not all created equal. The have been strategically stocked to bring supplies where they are needed. [adjust rest of document to match specific of department’s carts.]

#### ED Triage Cart/Ambulance Triage Cart

- These carts contain the most equipment. Here’s why: they are located where patient’s will be first arriving and in the worst shape. These locations do not have an everyday supply of trauma first aid equipment, so this is the reinforcement. Below is a picture of one of these carts with an index of their contents.

[Please insert a photo of department specific disaster carts. Here is one of ours as an example.][Fig f5-jetem-5-3-c1]

#### Pediatric Boxes

- Found in ED Triage Cart and Ambulance Triage Cart. These contain pediatric items related to both patient care and child life behavioral tools. Below is an example of a box and labeled contents:

[Please insert a photo of department specific pediatric disaster boxes. Here is one of ours as an example.][Fig f6-jetem-5-3-c1]

#### Alternative Hospital Entrance Cart

- This cart has the least amount of supplies. The majority of patients will NOT be coming through here.

[Please insert a photo of department specific disaster carts. Here is one of ours as an example.][Fig f7-jetem-5-3-c1]

#### ED High Acuity Area Cart

- Found in our high acuity pod within the ED. The key aspects here are the Job Action Cards and essential organizational materials (refer to Appendix C for more information on Job Action Cards). This cart contains comparatively less equipment for patient care because those resources are already in this treatment area.

[Insert a photo of department specific disaster carts.]

### Appendix N Call Out

**Note: Information in this appendix may be institution specific. Please evaluate content relevance and insert institute specific information as needed**

#### Objectives

At the end of this activity, the learner will be able to:

1. Understand the importance of waiting for a call before going to the hospital
2. Understand the standard call out procedure

In the event that an MCI occurs, **DO NOT** take it upon yourself to come to the hospital. There will be a call-out process. Remember that streets around the hospital will most likely be closed and parking will be at a premium. These issues can be minimized if staff DOES NOT self-deploy. If a call-out occurs during an MCI, it will be communicated via [your facility’s method for emergency communication] and will provide a phone number for you to call [substitute appropriate method for facility].

We learned through call-out drills conducted several times a year over various shifts that there should be a standardized format for responding to a call out. There were several calls during drills in which the staff member answering calls and voicemails could not identify who the caller was. It was also hard to decipher some or all of the information left on voicemail. This causes a delay in identifying which staff is available and when. In the event of an MCI, this is precious time we are losing. Below is the standardized process we developed for calling:

1. FULL NAME: including spelling. It is difficult for staff members who are answering calls & voicemails to decipher who is calling in if only a first name is given, especially if there are multiple staff in the department with the same name. The spelling will be helpful as all information will be logged and put into a grid to appropriately plan for staff.
2. DISCIPLINE: What is your role in the department? This will help staff identify when department needs are met.
3. WHEN ARE YOU AVAILABLE: Please include the date and time. This will help staff best fit the department needs. Remember traffic and parking will most likely be an issue, so plan ahead when arriving for your designated start time.
4. CALLBACK NUMBER: When it is identified when you will be needed at work, a staff member will call you to confirm the time.

Please do your best to minimize background noise when calling in. Also, speak in a normal tone of voice and speak slowly. Once again, **DO NOT** self-deploy to the hospital. Wait for direction to report to work.

### Appendix O Discharging

**Note: Information in this appendix may be institution specific. Please evaluate content relevance and insert institute specific information as needed**

#### Objectives

At the end of this activity, the learner will be able to:

1. Safely discharge patients both already in the ED and from the MCI
2. Recognize where to physically relocate discharged adults and children
3. Understand general discharge instructions and return precautions for common injuries from MCIs

If we find ourselves in a mass casualty situation, optimizing patient flow in and out of the department will be important. In a previous topic (See Appendix L), we discussed how to “purge” the ED of current patients--disposition people who are currently in the department at the time of an MCI as fast as possible. Additionally, we have to consider how to discharge the mass influx of patients we see from the MCI event itself.

#### Discharging people already in the ED

This is a refresher from a previous topic. If the patient does not need to be hospitalized and is stable, consider discharging them *now*. Refer them to urgent care or to their PCP. You should give them verbal discharge instructions (and maybe written discharge instructions) as you are rapidly showing them to the appropriate exit.

#### Discharging patients from the MCI

If we are dealing with penetrating injuries such as gunshot wounds, it will likely be fairly easy to identify which patients are uninjured and can safely go home. In the event of explosive injuries, however, we have to worry about delayed presentations such as blast lung. Pulmonary contusions from these injuries usually blossom over the first several hours but can present up to 48 hours after injury. A helpful tip: *if there is no hypoxia at 2 hours, these people rarely require intubation.*

#### Where do they go?

[This is institution dependent. Consider creating a “discharge lounge” somewhere in the hospital.] Patients should NOT wait in the ED because we will likely need all the space available.

#### Kids are not just tiny adults

Remember that unaccompanied pediatric patients do NOT get discharged to the waiting area. They all go to a Safe Child Area (refer to Appendix H for more information on child management).

#### Intoxicated patients

Consider discharging patients who are clinically sober. However, remember that if they are *not quite* clinically sober that they may have difficulty with the newly challenging outside world. A chair or a hallway bed might be a nice safe place for these individuals to metabolize (See Appendix L).

#### Return precautions and follow up

[Insert any references to pre-printed discharge instructions for patients from an MCI.] Some specific discharge instructions:

- Traumatic brain injuries (TBIs): assume lots of concussions. Can follow up with [institution specific TBI clinic, or otherwise]
- Ruptured tympanic membranes (TMs)s: ofloxacin drops twice a day for one-week, dry ear precautions and otolaryngology follow up in 1–2 weeks [or substitute for regional practice]
- Open wounds: may need delayed primary closure due to high risk of infection (especially blast injuries, shrapnel, etc). Refer for follow up. Antibiotics recommended!
- Lung injury: tell ALL your patients to return if they are short of breath
- Remember to encourage people to seek help for any psychological stressors they may have experienced during this event

### Appendix P Operating Room (OR) Prioritization

**Note: Information in this appendix may be institution specific. Please evaluate content relevance and insert institute specific information as needed**

#### Objectives

At the end of this activity, the learner will be able to:

1. Consider who will make decisions about what patient goes to the OR and in what order in the case of an MCI
2. Prioritize injuries needing timely OR intervention in penetrating trauma--abdominal/junctional injuries, then chest injuries, then orthopedic/head injuries

In the event of an MCI, we must decide with our trauma surgeons who goes to the OR and in what order. In an ideal situation, we would have a spare trauma surgeon in the ED to prioritize cases and a senior surgery resident staying in the ED to do admit orders. In reality, all trauma staff will likely be in the OR initially.

In this case, the ED physicians will need to make the OR prioritization decisions. [Consider creating a multidisciplinary team to create guidelines for how to prioritize these patients in your institution]. We have developed some general guidelines with our trauma surgery colleagues for how to determine in which order our patients go to the OR based on the mechanism of injury.

#### Penetrating injuries (ex: mass shooting)

- Abdomen/junctional: Very little can be done in the ED for these injuries (other than packing and pressure), so these will be taken preferentially to the OR.
  ○ Your focused assessment with sonography in trauma (FAST) exam is key here! Repeat and reassess as needed.
  ○ **Junctional** = junction of the torso and something else (neck, axilla and groin). These areas have big blood vessels not amenable to standard tourniquets. [insert information about junctional tourniquets if they are available in your facility]
- Chest: Most can be temporized or treated in the ED with decompression, chest tubes and intubation. Most penetrating trauma to the chest NEVER needs to go to the OR.
  ○ Remember: in an MCI we do NOT do resuscitative thoracotomies [insert information about retrograde endovascular balloon occlusion of the aorta (REBOA) if that this done in your facility] (takes time and resources away from the many other patients requiring our care).
- Head/ortho: These will be taken to the OR last.
  ○ Head injuries may have poor prognoses regardless of whether they go to the OR
  ○ Orthopedic injuries should be stabilized with reduction, control of the bleeding, washing out the wounds and giving prophylactic antibiotics (Refer to Appendix AB^‡^ for more information on temporizing for the OR)

#### Blunt trauma (or mixed blunt and penetrating, such as blast injury)

These are more complicated, so we do not have a clear-cut order of OR treatment for these injuries. We will likely need to rely heavily on patient stability and our surgery colleagues to help figure this out. What we do know is that as opposed to penetrating injuries, blunt trauma to the abdomen can likely wait to go to the OR because solid organ injuries are often managed conservatively^1^. Think about interventional radiology (IR) in these cases as well!

- Abdomen: Most commonly affected organs are spleen > liver > small/large intestine.
  ○ Signs of blunt abdominal injury: bruising, seat belt sign, ecchymosis on abdomen
- Chest: Think about the mechanism to determine what is likely injured. Think sternal fractures, flail chest, hemo/pneumothorax, aortic injury and pericardial effusions^2^.
  ○ Use a chest radiograph or ultrasound to help find these injuries.
  ○ If available, angiography can identify and classify vascular injuries (arterial versus venous).
  ○ 90% of most serious blunt cardiac injuries are lethal within minutes and these patients will likely die before arrival.
  ○ Treatment is mainly supportive and antiarrhythmics. Few cases require surgery or angiography.
  ○ Consider permissive hypotension to stabilize any clots that have formed.^3^

### Appendix Q Passive Security

**Note: Information in this appendix may be institution specific. Please evaluate content relevance and insert institute specific information as needed**

#### Objectives

At the end of this activity, the learner will be able to:

1. Describe the differences between active and passive security, and when each is utilized
2. Engage as an active member of our workplace’s security force

#### Passive vs. Active Security during an MCI

During an MCI, the safety of victims and responders is still an issue once inside the hospital. Threats and hazards will continue to evolve and recede. New risks can emerge. Dangerous people, diseases, and materials can still find their way through the doors if allowed, and it is critical we maintain awareness of who is entering and why.

**Remember**: Protect the house. This is where the concept of active and passive security comes in:

- **Active security**: High-profile. Based on overt security systems such as external security forces, security/access checkpoints, mobile police/guards, locked or blockaded non-essential access points.
- **Passive security**: Low-profile. Based on program design, warning systems, building/work space design, community participation and education, or anything that serves to mitigate potential threats.

*“If you see something, say something.”*

- Who does this apply to? **Everyone!** Even if your role is primarily clinical, we can all play a passive security role. You are already taking the first step in passive security by assessing and developing your own preparedness for disaster response!
- In MCIs, we are all a part of a larger passive security framework; it is our job to **ensure we know who comes in and out of our secured space**. By respectfully, but confidently, asking for identification and role of any unknown person we encounter, we can act to identify those who do not belong or who may pose a threat.

*“Hello, you’re in a restricted area. I see you don’t have a badge; can I help you find where you are trying to go?”*

Remember, these principles apply to everyday life here as well.

### Appendix R Evidence Preservation

**Note: Information in this appendix may be institution specific. Please evaluate content relevance and insert institute specific information as needed**

#### Objectives

At the end of this activity, the learner will be able to:

1. Understand that all patient belongings need to be treated as evidence
2. Recognize that patient care comes first before adherence to evidence preservation guidelines
3. Identify what would be considered evidence and who makes that determination
4. Identify process for collection of evidence

The most important thing to remember about evidence collection and preservation during any MCI is that ANYTHING may be evidence. Clothing, particles on the clothing, cell phones, jewelry… ANYTHING.

Collect everything you can. Never promise that the patient will get items returned to them because that is determined by whoever is doing the investigation for the MCI itself. If you are cutting anything off of a patient, remember to avoid any entrance points (rips, tears, presumed blast injury sites, etc). Investigators may use the characteristics of the damage to reconstruct what happened.

Bag all items in paper bags and label with patient name (if known) and plain language of what the bags contain (Ex: black jacket with left sleeve cut, no contents in pockets). Please do not write what you think happened (Ex: hole from gunshot, hole from knife, etc.)

We understand that heavy patient care demands will make following the standard policies for evidence difficult to follow. If law enforcement or other hospital personnel are available to collect evidence, give it to them. If not, **place all items under the cart the patient is on**. Make sure to communicate what belongings are present if any transfer of care occurs. Document what belongings are present as soon as possible. No one expects perfect adherence to the evidence collection policy under an MCI situation. Patient care comes first.

### Appendix S Family Reunification

**Note: Information in this appendix may be institution specific. Please evaluate content relevance and insert institute specific information as needed**

#### Objectives

At the end of this activity, the learner will be able to:

1. Establish a plan for how to reunite patients and their families
2. Consider logistical challenges that may be encountered

Once word of the event makes its way out into the community and media, people will flock to the scene or hospital to search for loved ones. This complicates matters by delaying emergency responders, heightening emotional responses, and further exposing those involved to media, spectators, and possibly additional threats of violence.

Other hospital staff [insert specifics] will be tasked with matching patients to their loved ones. Very few visitors will be allowed in the ED. Most reunification will happen in the inpatient wards or the [predetermined discharge area].

Actions that frontline ED staff can do to make it easier to identify patients and get them reunited with their loved ones:

1. Let the coordinator register them in [EMR]
2. Preserve potentially identifiable items such as ID, clothing, jewelry, etc.
3. When the dust settles, note identifiable features (tattoos, piercings, scars)

### Appendix T ^*^Coordinator Supplies

**Note: Information in this appendix may be institution specific. Please evaluate content relevance and insert institute specific information as needed**

#### Objectives

At the end of this activity, the learner will be able to:

1. Determine if necessary supplies are available
2. Understand how to use these supplies during an MCI
3. Identify 3 supplies that will be needed in an MCI

Set up for any MCI type of event will be hectic. There are not a lot of supplies that coordinators will need, but the supplies that they will need are vital.

- DOE TAGS: These will be stored with the rest of your facilities disaster supplies. Note they are separated into pediatric tags and adult tags [adjust for how they are organized in your facility]. If you are responsible for arriving patients, make sure to have both tags readily available. Also, be aware of where extra printer cartridges are.
- PEDIATRIC PATIENTS: [If your facility requires additional bands for pediatric patients (lab draw, radiation exposure, etc), make sure to have these available as well.]
- PEN & PAPER: Sounds basic but is vital.
- PPE: For arrival locations, use all departmental PPE supplies including gown, gloves, masks and booties. If you are unsure about any of these supplies, please ask [appropriate supervisor]. Refer to Appendix K for more information on personal protective equipment.

### Appendix U ^*^Answering Phones

**Note: Information in this appendix may be institution specific. Please evaluate content relevance and insert institute specific information as needed**

#### Objectives

At the end of this activity, the learner will be able to:

1. Determine the appropriate response to phone calls inquiring about missing family or loved ones
2. Identify scripting to answer calls quickly & efficiently

During an MCI, phones will be ringing constantly. Family, friends, media, concerned strangers and co-workers will be calling for different kinds of information. Calls will need to be handled quickly, efficiently and *correctly*.

Below are some scenarios and scripting to use:[Table t2-jetem-5-3-c1]

**Table t2-jetem-5-3-c1:**

Scenario	Message to Convey	Script
***Family of Patients in ED PRIOR to MCI***		
Still in ED [and confirmation of presence in the ED is allowed according to hospital policy]	Reassure family their loved one is being well cared for and take a message to pass on to patient. If you would typically give the call to the RN or patient, take a message (assuring the family that the call will be returned *as soon as possible*).	“Your loved one is still in the Emergency Department and is being cared for by our staff. I would be happy to take a message and pass it on as soon as possible.”
Admitted [and confirmation of presence in the ED is allowed according to hospital policy]	Reassure family the patient has been admitted to the hospital	“Your loved one has been admitted to the hospital and is being cared for by our staff. I would be happy to take a message and pass it on as soon as possible”
***Family of Unaccompanied Minors from the MCI***		
Child has been identified & ***still in ED*** [and confirmation of presence in the ED is allowed according to hospital policy]	Confirm the child is here & being cared for. Get someone here to be with child via the family reunification center.	“Your child is here & safe. He/she is currently being treated by our staff. When you arrive at the hospital please check in and we will be happy to reunite you.”
Child has been identified and ***discharged*** [and confirmation of presence in the ED is allowed according to hospital policy]	Reassure child is here & safe. Get them to the family reunification center to pick up child.	“Your child is here & safe. He/she has completed treatment and is currently with one of our staff members. When you arrive at the hospital please check in and we will be happy to reunite you.”
Child ***not*** identified or we are not allowed to disclose information due to hospital policy	Direct the call to the contact person in charge of family reunification for the hospital	“I’m sorry. I do not have a child by that name currently. We are still working on identifying all of our patients. Can I transfer you to [appropriate place to send that call] to provide your child’s information to?”
***Family/Friends of Identified Adults from the MCI***		
Patient still in ED [and confirmation of presence in the ED is allowed according to hospital policy]	Assure them that family member is being well cared for & take a message to pass to the patient when time allows	“Your loved one is here & is being cared for by our staff. I would be happy to take a message and pass it on as soon as possible.”
Unidentified MCI Patients	Direct the call to contact person in charge of identifying patients	“I’m sorry. I do not have a patient by that name currently. We are still working on identifying all of our patients. Can I transfer you to [appropriate place to send the call] to provide your loved one’s information to?”

### Appendix V ^†^Patient Belongings

**Note: Information in this appendix may be institution specific. Please evaluate content relevance and insert institute specific information as needed**

#### Objectives

At the end of this activity, the learner will be able to:

1. Understand how to handle patient belongings
2. Understand procedures surrounding weapons or dangerous items found in patient belongings

#### Patient belongings are to be kept with patients whenever possible

During an MCI, our standard procedures for managing patient belongings by cataloging them would take far too long. However, we cannot simply ignore patient belongings because these may be important in identification of unknown patients or in the eventual investigation of the event. You will not be able to, or expected to, perform full chain-of-evidence protocols during the event itself. As such, keeping the patient’s belongings with the patient is the simplest solution.

Large belongings bags will be made available in [location]. All patient belongings, including things like clothes that are cut off, should be placed in these bags, labeled with the current [EMR] identification tags, and then be kept with the patient. If patients are on a gurney, put the bag at the foot of the bed or below the gurney, or in their arms if they are in a chair. When taking someone to the operating room or an inpatient floor, please check under the gurney and make sure to send the bag with the patient (Refer to Appendix AB^‡^ for more information on temporizing for the OR).

#### If weapons or dangerous items are found: [adjust for your facility’s policy]

1. Ensure staff safety. Make sure all staff members in room are aware of weapon’s existence.
2. Notify security. Make sure the dispatcher knows if the patient still has access to the weapon.
  - If the patient has access to the weapon, security and/or law enforcement will arrive quickly.
  - If patient is not able to access the weapon and ED staff is safe, they will still come for guns and large knives but be aware they will likely be stretched quite thin.
3. If patient unable to access weapon, separate weapon from patient (if can be done safely) and [follow institution-specific guidance].
4. If you are concerned there may be a bomb on the patient, follow the standard procedures;
  - All staff evacuate immediately! Staff safety first, then patient safety.
  - Notify security and they will take it from there (Notify 911 and activate bomb threat for hospital).

### Appendix W ^†^Reprocessing

**Note: Information in this appendix may be institution specific. Please evaluate content relevance and insert institute specific information as needed**

#### Objectives

At the end of this activity, the learner will be able to:

1. Recognize when to deviate from standard reprocessing procedures
2. Understand the importance of monitoring equipment supplies and anticipate needs

In the middle of an MCI, we will be dealing with a large number of critically ill or injured patients, many of whom may require intubation. As such, reprocessing will still need to be done and the procedure will be the same as normal unless directed otherwise by the charge nurse. We will likely be going through a lot of laryngoscope blades so it is imperative that these are dealt with as soon as possible.

As a reminder: reprocessing for laryngoscopes

[Insert steps here]

Obviously, our typical processes will get overwhelmed in an MCI. The [appropriate role such as Lead ERT] will work with the charge nurse to determine when and how we will vary from standard operating procedures. As soon as you notice supplies running low, let the [appropriate role such as Lead ERT] know so he or she can plan ahead.

For all of the other, less urgent utensils, you can still follow your normal procedures for reprocessing. Whenever you have a free moment quickly check the dirty utility rooms in your areas for accumulating tools and utensils. Let your lead know if the utensils are just piling up and you won’t have any time to run them upstairs. Otherwise, be vigilant and observant. If you can, keep an eye on what is being used and try to predict what will be needed. Anticipate we will be placing lots of chest tubes, intraosseous devices and suturing lots of wounds. Know where extras are stocked. Notify the [appropriate role such as lead ERT] early when we start to run low. Send non-disposable tools for reprocessing quickly.

### Appendix X ^†^Trash

**Note: Information in this appendix may be institution specific. Please evaluate content relevance and insert institute specific information as needed**

#### Objectives

At the end of this activity, the learner will be able to:

1. Distinguish the differences between the three possible types of trash that can accumulate during an MCI
2. Understand how to manage decontamination area trash, biohazard trash, and normal trash during an MCI

Throughout an MCI, waste, ranging from wrappers to soiled gauze, will constantly accumulate. As ERTs, one of your priorities is the management of patient belongings and keeping them separate from the trash. If you find that the trash in your room needs to be changed, call housekeeping and let them know because management of waste is one of their primary responsibilities in this event. As such, they will be as busy as you are so be patient with them, but do not be afraid to call again if you haven’t seen anyone for 15 minutes. In a pinch you can always change the trash yourself, although this is less than ideal because housekeeping will still need to come and pick up the bag.

All waste generated by the decontamination team for a chemical, biological, or radiological decontamination should be kept together in the decontamination area to be dealt with as hazardous materials. Belongings in this area should be separately bagged and labeled to ensure that nothing is accidentally thrown out with the trash. By the time patients get into the ED, all decontamination will be done and they should not require further precautions be taken due to the reason they were decontaminated. However, since mistakes can be made, you should always be wary of unusual waste in these events.

Infectious waste such as blood and bodily fluids will be an issue. Anything that is supersaturated needs to go into the red biohazard bins found in every dirty utility room. Small red biohazard bags, stocked in the utility rooms, should be used to transfer the soiled items without dripping waste on the floor. Not only does this prevent the spread of infectious material, it also keeps the floors clean and clear of trip hazards.

Good waste management in the ED will smooth out our MCI operations. Too much waste accumulation can make turning over rooms difficult and time consuming. Because time is our most valuable asset in these situations, you should stay on top of waste as best as you can. It is not your first priority, but you should still do your best to keep the ED clean!

### Appendix Y ^‡^Biological Agents

**Note: Information in this appendix may be institution specific. Please evaluate content relevance and insert institute specific information as needed**

#### Objectives

At the end of this activity, the learner will be able to:

1. Distinguish biological attacks from other mass casualty incidents
2. Identify signs of biological agents

While bombs, active shooters, and chemical attacks are all very likely to cause our ED to gear up for an MCI, biological attacks are much less likely to do so. A truly successful biological attack may not be noticed for weeks and would not cause a large influx of patients requiring SALT Triage (refer to Appendix AF for more information on SALT triage.) Instead, over the course of a few weeks, you would notice an increase in specific symptoms and diagnoses, something far out of the ordinary. An example would be an unusual diagnosis becoming more and more common over the course of a few days. However, not all cases will initially present uniquely. The first symptoms of Ebola are flu-like symptoms that we see all the time, and could easily be mistaken for something non-threatening, especially if the person had not been traveling abroad. Therefore, the best way to identify a biological attack is vigilance!

Unusual and strange cases coming through the ED should be reported to the Charge Nurse and Attending Physician, with the on-call Infectious Disease Physician also available for advice on isolation and treatment. Since these events will be affecting many hospitals in the area, coordinating with the Department of Health (DOH) is paramount for recognizing the breadth of the event. Our infection prevention department already maintains contact with the DOH and can help pass along vital information to the investigating epidemiologists. Furthermore, the DOH may have recommendations for isolation or treatments depending on the specific nature of the agent.

A true MCI with biological agents would be something more like an explosion at a lab building, requiring decontamination of all injured people before they are brought into our ED. Another example would be a deliberate act, such as white powder being spread over a large group of people. All of these attacks would result in the same response from the ED: an MCI and decontamination event. If the patient is thought to be infectious after decontamination, the patient should be placed in isolation based on the nature of the agent. Many infectious diseases are not immediately contagious because they must incubate for a period of time before person to person transmission is possible. As such, proper decontamination should prevent transmission of most diseases directly after the exposure event. While most biological agents are not able to be absorbed through the skin, there is a small group of toxins that are able to be absorbed through the skin. Because of agents like these, you should always be vigilant when dealing with potential biological contaminants.

These ‘true’ biological MCIs due to accidental or deliberate exposure events would be very similar to a chemical or radiological MCI in that they would all start with decontamination, after which they would proceed like any other MCI. The real danger in biological attacks is the widespread, dangerous outbreaks which affect whole cities and regions. On the front lines in the ED, you are in the perfect position to observe and notice these outbreaks as they emerge!

### Appendix Z ^‡^Airways

**Note: Information in this appendix may be institution specific. Please evaluate content relevance and insert institute specific information as needed**

#### Objectives

At the end of this activity, the learner will be able to:

1. Determine when advanced airway establishment should be prioritized during an MCI
2. Review the basic logistics of airway management (ie, tools, medication, ventilators, etc)

During a mass casualty incident, lots of sick people will roll through and many of them will need advanced airways. Our standard operating procedures will quickly fall to the wayside. In addition to the usual indications for intubation, any patients going to the OR should be intubated in the ED prior to being sent upstairs to keep the OR process running smoothly. [insert information about specific airway equipment for MCI available in your department, such as disposable laryngoscopes]

#### Logistics

Video laryngoscope blades have a prolonged reprocessing time (3.5–4 hours.) Consider saving them for inexperienced users or the anticipated difficult airways. Plan on using direct laryngoscopy. We have many laryngoscope blades in varying sizes. Also, remember we don’t actually need to *see* the vocal cords to manage an airway. Use the endotracheal tube introducer (bougie) or a supraglottic airway. Remember digital intubation? No tools needed for that.

***Note (particularly for [ERT]): If you notice we are running low on equipment, please tell the [appropriate role such as Lead ERT] ASAP*** **.**

#### Medication

Frequently used intubation medications will be released from the automated dispensing cabinets for quicker access (think pharmacist with a bucket of meds). We are stockpiling the medication that does not need refrigeration. The only paralytic not needing refrigeration is vecuronium. You can use the usual intubation meds, or consider intramuscular (IM) ketamine 4–5 mg/kg in a rapidly crashing patient without access who is requiring immediate intubation, chest tube placement, etc.

#### Ventilators

**Quiz:** How many respiratory therapists are in the building at one time? Answer: not nearly enough. Can you set up a vent? If able to, put patients who are going expediently to the OR immediately on transport ventilators. We have about [insert number of ventilators] ventilators in the hospital. Speak up if we are getting low. The hospital has back up plans, but they take time.

If running low on ventilators, consider Y-tubing to connect two patients of similar size to one ventilator. Don’t forget to DOUBLE the tidal volume in this situation.^1^,^2^

**Bagging:** In a worst-case scenario, we may require extra hands for bagging. This would be a great job for a medical student. In a very dire situation, consider having non-clinically trained staff members bag them. Either way, coach the person on squeezing the resuscitation bag -way and at an appropriate rate (by age and condition), since they are very likely to over-ventilate in this anxiety-producing situation.

**Suction:** Working suction, or lack thereof, can make or break airway management. ERTs need to be on top of suction setups. Many chemical agents cause obscene amounts of secretions.

### Appendix AA ^‡^Bleeding Control

**Note: Information in this appendix may be institution specific. Please evaluate content relevance and insert institute specific information as needed**

#### Objectives

At the end of this activity, the learner will be able to:

1. Identify the tools available to assist with hemorrhage control
2. Understand the basic approach to controlling hemorrhage

In many MCI events, there will be a number of victims who require immediate treatment to stop life-threatening hemorrhaging. Profuse bleeding is a common companion of major trauma. Controlling bleeding is crucial to volume retention and preventing hypovolemic shock, and doing it properly can make all the difference. Wear PPE! Gloves, gown and mask with eye protection. Refer to Appendix K for more information on PPE.

The most commonly accepted and utilized methods of hemorrhage control are direct pressure, elevation, and tourniquets. All three should be used in order to attempt to control bleeding. As the amount of volume loss increases or the severity of the wound dictates, you may need to employ all three methods.

#### Direct Pressure

Direct pressure is the most commonly used and effective bleeding-control technique, and it usually controls most external bleeding. What is direct pressure? One or more fingers (as few as possible) placed directly at the site of bleeding. Press until the bleeding stops. Despite the simple name and simple technique, direct pressure is often executed by placing abdominal pads and layers of gauze loosely on a wound. These bandages become blood soakers and do nothing to abate ongoing bleeding, they only cover it up. Gauze can be used effectively here if it is soaked with tranexamic acid (TXA) or packed in a way to place pressure on a specific point.^1^

#### Elevation

The second method used to control bleeding is elevation of an injured extremity, which is most often done along with direct pressure. The goal is to raise the extremity above the level of the heart to decrease circulation to that area. A sling or some other way of maintaining elevation may be used but be sure to keep the injury site above the level of the patient’s heart.

#### Tourniquets

The disaster carts will contain many additional tourniquets to supplement our stock. Our department uses the [insert stocked brands here]. Proper application is essential. The overstated concern of the complications of tourniquet use in the field contrasts with the experience in the operating room when tourniquet use occurs routinely for relatively extended periods.^2^ Accurate documentation of the time of tourniquet application is recommended, and in prehospital settings, is often written on the patient’s forehead or on the commercially available tourniquet on the provided tag.

#### Other Treatment Methods

In addition to the methods above, many commercial products have recently been developed for the prehospital setting that can help to control bleeding. Several were first used in the military/combat setting but have proved applicable elsewhere.

**Hemostatic gauze:** Several brands of hemostatic gauze are available. Our facility stocks [insert brand of gauze available or delete this sentence if none is available.] This agent works by absorbing the liquid (plasma) from blood, reducing clotting times. These products are available in a range of forms including granules and impregnated gauze. As a general guideline, hemostatic gauze should not be the primary treatment for wounds, and is recommended to be used after alternative methods including tourniquets fail or cannot be used. In the setting of life-threatening bleeding, however, this should be used liberally.^3^

**Tranexamic Acid:** TXA is an inexpensive medication FDA (US Food and Drug Administration) approved for short-term use in patients with hemophilia to reduce or prevent hemorrhage.^3^ However, **it can and should be used on an off-label basis for reduction and prevention of hemorrhage in trauma patients**. This is available and stocked in the [automated medication dispensing cabinet]. Intravenous TXA should be given as soon as possible, within three hours of injury, to all patients at risk of death due to bleeding. TXA is not effective if started after three hours. We anticipate this will be many if not all patients triaged to a red tag.^4^ Any MCI patient needing blood transfusion should receive TXA if it can be given within three hours of injury. During an MCI, clinicians will need to use their clinical judgment to determine need for transfusion. The adult loading dose of IV TXA is 1 g over 10 minutes and works best as soon as possible within the first 3 hours after injury.^4^ After the load, an infusion of 1 g over 8 hours should be given if resources allow. Pediatric dose is 10 mg/kg actual body weight. We fully expect our ED to use a LOT of TXA during an MCI [insert specific facility plans such as stockpiling].

#### Conclusion

Profuse bleeding is a common companion of major trauma and mass casualty. Controlling bleeding is crucial to volume retention, preventing hypovolemic shock, and preserving limited transfusion supplies. Using some simple and effective methods, along with some progressive new products, we can effectively treat and control significant hemorrhage.

### Appendix AB ^‡^Temporizing for the OR

**Note: Information in this appendix may be institution specific. Please evaluate content relevance and insert institute specific information as needed**

#### Objectives

At the end of this activity, the learner will be able to:

1. Understand how to replace volume loss with fluids and, more importantly, blood
2. Identify ways to stop blood loss such as direct pressure, tourniquets, pelvic binders and interventional radiology
3. Understand the importance of other interventions to buy time while waiting for the OR such as washouts, antibiotics and pain medications

In an ideal world, every patient requiring surgery during an MCI would be immediately whisked away to the OR for life saving procedures by our esteemed surgeons. Unfortunately, due to the mass influx of patients during an MCI this likely will not happen. Imagine you have a critically ill patient that you need to stabilize for possibly minutes or hours? Here are some things to think about:

#### (Re)fill the tank

If your patient becomes hypotensive, give them back volume. You can start with IV fluids, but ultimately they need blood. Remember that a tension pneumothorax, hemothorax, etc. can also cause hypotension so reassess and decompress the chest if needed!

#### Stop the bleeding

This is covered in a separate topic (refer to Appendix AF^‡^ for more information on SALT triage). If you can see a source of bleeding, stop it! Hold pressure with smallest surface area possible, think a fingertip! This may be a great job for a medical student, ERT or even a family member. You can also pack the wound if you can’t find the exact source of bleeding in an attempt to induce tamponade.

#### Tourniquets

Tourniquets are life saving measures that can hold off blood loss from injured extremities. Ideally, we remove these as soon as possible to prevent ischemia. However, if we are awaiting placement in the OR, tourniquet time can be a lot longer than we thought. Life over limb!

#### Pelvic Binders

A fractured pelvis is an open structure that can hold 5L of fluid or more, possibly your entire blood volume^1^. Pelvic binders go over the greater trochanter, the widest part of the hips, and can decrease retroperitoneal bleeding significantly^2^. In a pinch, a sheet will also do the trick^3^. When in doubt, put the pelvic binder on! Once the binder is on, there are very few reasons to remove it. Skin ischemia can happen, but weigh that risk with the clinical scenario.

#### Interventional Radiology (IR)

Some vascular injuries in the pelvis can be fixed with IR embolization. Angiography treats arterial bleeding, not venous bleeding. Venous bleeds are more common in general, but arterial bleeds are increasingly likely in elderly patients^4^. Call your consultants to see if your patient would be an IR candidate.

#### Wash out and Antibiotics

Injuries from an MCI are likely very contaminated. Think about shrapnel, dirt, and bullets flying through things and possibly even other victims before getting to your patient. Not a pretty picture. Make it a priority to try to clean these wounds out as best as possible; this may be a great job for our ERTs or medical students. Orthopedic injuries and open fractures will be last on the list of injuries to be fixed in the OR because these can wait for much longer periods of time. Keep patients stable until then by getting the wounds washed out and antibiotics in their system.

#### Pain medications

Treat your patient’s pain. In the unfortunate situation that your patient will likely not make it to the OR soon, remember to provide appropriate analgesia. Consider oral and other parenteral routes such as sublingual and intranasal medications in these patients to conserve more limited and resource-intensive intravenous medications.

### Appendix AC ^‡^Infusing Fluids

**Note: Information in this appendix may be institution specific. Please evaluate content relevance and insert institute specific information as needed**

#### Objectives

At the end of this activity, the learner will be able to:

1. Identify alternative methods to rapidly infuse fluids safely
2. Identify three ways to rapidly infuse fluids
3. Identify three potential issues to troubleshoot when fluids are not infusing

During an MCI, it is highly likely, if not guaranteed, that you will have to give patients fluids to replace volume lost. Be prepared to do this quickly and possibly in ways you may not practice on a “normal day.” As a general rule of thumb, we will continue to operate with equipment as long as it is available, including a [insert specific rapid infuser], pressure bags and IV pumps. Once these supplies run out, staff may need to get creative. You can “hand squeeze” fluids or ask another staff member to assist if they are able. Remember, extra hands may not always be available to help! You can also use a manual BP cuff on an IV bag as well.

It is also important to consider what your access is when rapidly infusing. Large bore IVs (18G or larger) and IOs are preferred over a triple lumen due to size and length of the catheters used. When using an IO, the fluid will need some kind of pressure to infuse, whether it is a pressure bag, IV pump, or someone physically squeezing the bag.

If you are having difficulty getting fluid to infuse, make sure the lines (both the IV tubing and any connection tubing) is not clamped. Look at the roller clamps, remembering that the [rapid infuser] tubing has multiple clamps, and so does blood tubing! Make sure all blue slider clamps are open as well. Double check to make sure there are no kinks in the tubing, and make sure nobody is standing on the tubing! There could also be a kink in the IV catheter which you cannot see, so as a last resort, try to adjust the IV catheter.

The bottom line is to use whatever means necessary to *safely* deliver the necessary amounts of fluids to replace the volume lost.

### Appendix AD ^§^Crisis Standard of Care

**Note: Information in this appendix may be institution specific. Please evaluate content relevance and insert institute specific information as needed**

#### Objectives

At the end of this activity, the learner will be able to:

1. Explain why crisis standard of care is important
2. Identify basic differences between day-to-day care and standards of care in a crisis situation
3. Feel more comfortable abandoning usual practices in order to be efficient and care for the most patients possible

In a mass casualty event, we don’t take care of people the same way we normally do. We must utilize limited resources to benefit the greatest number of people possible^1^. We can quickly become overwhelmed if we adhere to the same standards of care as day-to-day practices. In fact, the Institute of Medicine says that the crisis standard of care is not a choice but a duty and that failure to adopt this standard would likely result in greater death, injury, or illness^2^. In brief, this means focusing on the care of the population rather than the individual patient.

In the setting of an MCI, here are some basic guidelines

- No chest compressions
- No thoracotomies
- If pulses are lost, stop
- Ask how bad of a head injury is too bad

A lot of the decisions above may sound difficult, and they will be. You should rely on the attending physician’s judgment. Lean on them and their years of expertise! That’s why they get paid the big bucks!

#### When in "completely overwhelmed" mode

Within the first few minutes to hours of an MCI, we will have a large influx of critically ill patients. During this time, we need to be as efficient as possible and rely heavily on bedside examinations rather than diagnostic testing. This means:

- No x-rays
- Lab- ONLY type and cross (so we can get to type specific blood ASAP and conserve O− and O+)
- Lots of eFAST (focused assessment with sonography in trauma)--point-of-care ultrasound for pneumothorax, hemothorax, free fluid, and tamponade
- Use TXA liberally
- Can’t get an IV? Drill an IO
- Extremity injuries: Use tourniquets (write time placed somewhere), splints for hemostasis only (femurs bleed a lot) and straighten out mangled limbs to get pulses back

#### Documentation

Documentation will be challenging, and extremely limited during an MCI. [Insert institution-specific information about documentation such as using rudimentary paper charting-perhaps just a scrap of paper with the “Doe” name on it on the patient’s gurney where we can list the injuries that have been identified.]

#### MCI criteria for invasive procedures

- Chest tube: decreased breath sounds + unstable vitals. You likely won’t have a chest x-ray for confirmation. Decompress that pneumothorax or hemothorax to stabilize the patient. Don’t forget finger thoracostomy.
- Intubation: for patients not protecting their airway or “sort of” not protecting their airway. It may be easier to just intubate or place a supraglottic airway than to reassess frequently.
  ○ May need to call on others (non-ED hospital employees, scribes) to bag the patient
  ○ Remember ventilators will be limited! In Las Vegas, they had to double up (two patients per ventilator, double the tidal volume)
- Blood transfusion: Transfuse if hypotensive and tachycardic. Don’t forget TXA.

#### Other helpful tips

- Think about antidotes for toxicological issues
- Minimize IV drips. Ketamine will be the go-to sedative due to its hemodynamic properties and lack of adequate staff to monitor continuous infusions.
- There may be a time when we re-use products that we normally sterilize or throw away if resources are tight (C collars, tourniquets, etc.)

#### When in "starting to catch up a little" mode

This will likely occur several hours into the MCI. At this point the most critically injured patients should already be up in the OR. Start catching up a little bit and re-assessing all of our patients. In general, this means:

- Start getting labs
- Give type specific blood
- Re-assess your patients: does this patient need to go to OR now?
- Did I mention re-assess your patients? Patients who were initially stable won’t necessarily stay that way
- Extremity injuries: now you can splint, reduce, consult as able (surgeons should be in the OR). Wash out open fractures, give antibiotics to delay the OR, and discharge whatever we can.

Remember that even in MCI situations palliative care is always appropriate! If it seems like nothing can be done for that devastating injury, don’t forget to think about pain management and doing what we can to keep the patient comfortable. When in doubt, remember that the goal in a mass casualty situation is to help the highest number of people possible.

### Appendix AE ^§^Blast Injury

**Note: Information in this appendix may be institution specific. Please evaluate content relevance and insert institute specific information as needed**

#### Objectives

At the end of this activity, learners should be able to:

1. Recognize the wide variety of injuries that can present as a result of a blast injury or explosion.

Bombings and other blast injuries are common causes of mass casualty incidents. Specifics of when, where, and how care will be provided depend on the situation and number of casualties.

This is not meant to be a full review of blast injuries – just an overview to refresh your memory or pique your curiosity. Injuries can come from the blast wave itself, from objects being thrown at victims, the victim being thrown, crush injuries, or burns. Blast injuries come in several flavors--blunt, penetrating, crush injury, burns, pressure waves, etc. Most deaths come from head injury, but lung injury will utilize lots of hospital resources. Explosions or blast injuries can lead to a wide variety of injuries to nearly all organ systems.

#### History

In an MCI, we won’t be gathering lots of history from patients. However, a few details will help us predict how traumatized a patient is. Was the patient indoors or outdoors? Patients who are indoors may have been exposed to higher pressures. Were they crushed? How close to the explosion was the patient?

Primary survey (Because penetrating trauma is common in blast injury, we suggest using MARCHE: massive hemorrhage, airway, respiratory, circulation, and hypothermia, instead of ABCDE: airway, breathing, circulation, disability, and exposure.)

#### Massive Hemorrhage

Massive hemorrhage kills patients quick and must be stopped as soon as possible. Look for bleeding and stop it with dressings, tourniquets, etc. For speed and efficacy, place a hasty *tourniquet*. That means place a tourniquet as proximal and tight as you can.

#### Airway

Intubate early. Re-assessments will be difficult with limited staff. Avoid succinylcholine in patients crushed or trapped for many hours. Confirm airway placement with end tidal carbon dioxide monitoring, exam, and perhaps ultrasound (no chest x-ray right away, it’s too slow). Be ready to suction frequently because pulmonary edema from blast lung may develop. Don’t forget sedation and pain control!!

#### Respiration

Blast lung acts like pulmonary contusion and a thermal burn; it blossoms over the first several hours. Victims likely inspired a lot of heated air. Most severe blast lung patients need to be on a ventilator for 4–7 days. Ventilator supply will be an issue.

Expect hemothoraces and pneumothoraces. Decide on chest tubes based on physical exam and point-of-care ultrasound in MCI setting. Consider finger thoracostomy for emergent decompression. (scalpel, Kelly clamp or finger, dressing). Do not obtain chest radiographs right away when overwhelmed in an MCI.

Use lung protective ventilation in blast injury patients (tidal volume 6ml/kg ideal body weight). Watch non-intubated pts. carefully for signs and symptoms of blast lung such as tachycardia, cyanosis, cough, hemoptysis.

#### Circulation

Assume shock is hemorrhagic, and from penetrating and/or blunt trauma. Give tranexamic acid (TXA) liberally, 1g bolus followed by an infusion if resources allow. Replace volume. Decide on blood transfusion based on vital signs and amount of bleeding (ie, no labs, no point-of-care labs in early phases of MCI).

Perform bedside ultrasound to look for sources of bleeding in the abdomen and chest. Be prepared to repeat often. Reduce angulated long bones to improve perfusion. Track chest tube outputs. Transfuse as clinically indicated.

Consider systemic air embolism. Signs and symptoms include sudden blindness, focal neurological deficit or loss of consciousness, chest pain, livedo reticularis (reddish-blue mottling), tongue blanching, pharyngeal petechiae, hemoptysis.

Initial treatment is high levels of oxygen. If on vent, keep pressures and volumes low, turn patient to left lateral decubitus or Trendelenburg positioning to prevent cerebral embolization. If unilateral lung injury is present, keep injured lung lower than left atrium. Hyperbaric oxygen as a last resort but will likely be difficult to accomplish in an MCI setting.

#### Head/Hypothermia

Head injury is a more likely cause of death than lung injury. If intubating for altered mental status, try to get a Glasgow Coma Scale score first. Pupil exam is complicated in these patients. Pressure waves can damage or rupture globes. If globe rupture is suspected, gently tape the lid closed and protect the eye with a shield.

If the patient has lateralizing neurologic signs, intubate then mildly hyperventilate. Give hypertonic saline if available. Obtaining a computed tomography (CT) is ideal but may not be feasible early in the MCI response. Consider expectant management (gray or black tag) for obvious devastating head injury. When able, head CT can help with prognostication. Spinal injuries will likely be found and managed later on in the response. Temperature needs to be managed, particularly if the incident or initial triage happens outside in colder weather. Hypothermia impairs coagulation. Use blankets and hot hats to preserve body temperature.

#### Everything Else

Fully expose the patient and look for penetrating wounds (shrapnel, etc), burns, dialysis access or medic alert bracelet. Beware of tiny wounds; there may be embedded shrapnel. Recall that all that shrapnel/fragmentation in or on the patient is evidence. Try to treat it as such, though patient care trumps legal and evidentiary concerns.

#### Secondary Survey

- **Head, eyes, ears, nose and throat (HEENT):** Look in ears for *tympanic membrane (TM) rupture* (especially in less injured patient). The TM is the most sensitive structure to pressure changes. This might present days later. Examine eyes carefully, looking for globe rupture, retrobulbar hematoma, or other serious ocular trauma.
- **Chest/cardiovascular:** Watch for sudden cardiovascular decompensation (see air embolism above). Consider thoracic eschar syndrome in patients with significant burns. Watch monitor for signs of hyperkalemia.
- **Abdomen:** eFAST (extended FAST US exam) will need to be done frequently in patients awaiting disposition from the Emergency Department. Examine for peritonitis because perforated viscus is common. CT of abdomen should be done when able because this will help the surgical team to triage patients to non-operative management.
- **Genitourinary:** Intubated patients will need urine output monitoring. Dark urine suggests rhabdomyolysis.
- **Ext/Skin:** Compartment syndrome is a concern. X-rays should be obtained when able. Fractures should be splinted for hemostasis and pain control. Irrigate open fractures and give antibiotics because OR will be delayed for these patients. Avoid primary wound closure since wounds are contaminated. Give tetanus prophylaxis as able.
- **Neurological:** If there is a sudden decompensation or vision change, consider cerebral air embolism.

Be observant of patterns and unique needs so the rest of the hospital can adapt strategy and prepare staff and equipment. Unexpected findings such as toxidromes, unusual foreign bodies found or obvious biological contamination can alert responders to potential chemical, biologic, or radiologic attack.

Early notification about special populations of patients can allow the lead physician and Incident Command arrange consultants, prepare specific levels of care, or specialized care such as intensive care, obstetrics, pediatrics, burn unit, etc.)

#### Green Tagged (Non-Urgent) Patient Care

##### Ear Injuries

Patients may not hear you well and not respond when called. Objective evidence of ear damage *may* predict lung and gastrointestinal injury. Anyone with fluid or blood leaking from ears needs oxygen sat monitoring (or at least spot checks). Observe these patients at least a couple of hours.

Vertigo will be a common complaint but is more likely to be due to traumatic brain injury than ear issue. Tympanic membrane ruptures will be common.

##### Blast Lung Injury (early)

Blast injury can present UP TO 48 HOURS after injury. Signs of blast lung include tachycardia, cyanosis, cough, and hemoptysis. Place staff in the waiting area to watch for signs of respiratory decompensation as patients wait to be seen. Re-triage as needed and change tag color as needed.

#### Patients who are more than 2 hours post-injury without evidence of hypoxia rarely require intubation for blast lung injury

##### Special populations

- Pregnant patient in 2nd or 3rd trimester – get Kleihauer-Betke, Rh. Transfer to labor and delivery for monitoring. Consult OB early.
- Kids: Tympanic membranes are less likely to rupture. Therefore, it is prudent to observe pediatric patients for a couple of hours for respiratory decompensation.

##### Discharge considerations

Wash wounds and refer for delayed primary closure because most will be too contaminated for primary repair. Fragments and shrapnel can travel through lot of things, and unfortunately people, before wounding your patient. Liberal use of antibiotics recommended.

Council patients on traumatic brain injury precautions. Also discuss strict return precautions for any respiratory symptoms.

### Appendix AF ^§^SALT Triage

**Note: Information in this appendix may be institution specific. Please evaluate content relevance and insert institute specific information as needed**

[Disclaimer: Several triage systems exist. This appendix focuses on SALT. People using this guide should adjust as needed based on the triage system their facility uses.]

#### Objectives

At the end of this activity, the learner will be able to:

1. Understand the process for triaging patients during an MCI
2. Identify the four main triage categories

Quickly establishing a level of organization is essential. The role of triaging patients to the appropriate level of care will be the responsibility of the [insert role such as ambulance triage RN]. The goal of triage is to quickly identify those victims likely to die within the first 60 minutes of the incident if they do not receive proper medical care. SALT, which stands for Sort, Assess, Lifesaving interventions, Treatment and/or transport is the four-step process for nurses and providers to manage mass casualty incidents. This system provides a framework of clear, simple steps that providers can use to bring order to chaos and help improve patient outcome.^1^

Patients will be triaged into four categories and will be given a physical tag that is color-coded. This process happens in Step 2, outlined below.

**Expectant/Deceased**

- Victim already dead or unlikely to survive given severity of injuries, level of available care, or both
- Palliative care and pain relief should be provided

**Immediate**

- Victim can be helped by immediate intervention
- Requires medical attention within 60 minutes for survival
- Includes compromises to patient’s airway, breathing, or circulation

**Delayed**

- Victim’s medical attention can be delayed at the priority of helping those in the *immediate* category
- Includes serious and potentially life-threatening injuries, but status not expected to deteriorate significantly over several hours

**Minor**

- Victim with relatively minor injuries
- Status unlikely to deteriorate over days
- May be able to assist in own care: “walking wounded”

Sorting patients into categories for immediate, delayed, or minor treatment saves lives and provides an effective structure to the initial, chaotic stages of an MCI.

[Consider inserting a picture of institution specific triage tags]

#### Adult Triage Algorithm: 2

[Insert graphic of algorithm being used at your institution]

Step 1: Sorting; Walk, Wave, or Still?

- Individual patient assessments will occur in a certain order based on a patient’s ability to either walk or wave in response to verbal stimuli.
- Those who are not able to walk or wave to verbal stimuli are assessed first. Those who are not ambulatory but are able to respond to verbal stimuli are assessed second. Ambulatory individuals are assessed third.

Step 2: Individual Assessment

- With the SALT system, assessment and lifesaving interventions go hand in hand.
- First, identify any immediate life threats such as major hemorrhage, tension pneumothorax/hemothorax, anaphylaxis, or occluded airway.
- When you assess a patient and find a life threat you should provide a lifesaving intervention as long as it can be accomplished quickly.
- If you find that a patient has massive hemorrhage, provide rapid bleeding control with a tourniquet.
- If a patient’s airway is closed, reposition the airway. If that patient is a child or infant, consider giving them two breaths.
- If you have the skill-set, it may be appropriate to provide needle decompression of the chest, auto-injector chemical toxin antidotes or other lifesaving interventions.

After this initial round of life saving interventions, it is time to move forward with tag application.

##### DECEASED/EXPECTANT (BLACK TRIAGE TAG)

Patients with injuries incompatible with life or without spontaneous respirations are triaged as deceased. If a patient makes no purposeful movement, has no peripheral pulse, is in respiratory distress or has uncontrollable major bleeding, and is felt to be unlikely to survive given the available resources, that patient should be tagged expectant. These patients should receive treatment resources only after the immediate (red-tagged) patients have been moved forward. Examples of expectant patients include head injury with exposed brain matter, carotid artery hemorrhage or burns to 90 percent of the total body surface area.

Assess the following:

- Adult patient is not breathing after opening airway.
- Child is not breathing after opening airway and giving two breaths.

##### IMMEDIATE (RED TRIAGE TAG)

Patients with severe injuries, but high potential for survival with treatment such as victims of tension pneumothorax; assess the following:

- Does the patient have a peripheral pulse?
- Is the patient not in respiratory distress?
- Is hemorrhage controlled?
- Does the patient follow commands or make purposeful movements?

A "no" answer to any of these questions, and provider judgment that the patient is likely to survive given the available resources, means the patient should be tagged immediate.

Immediate patients move forward to be treated first.

##### DELAYED (YELLOW TRIAGE TAG)

Patients with serious injuries, such as a long bone fracture, that will require eventual forward movement to definitive treatment, but not immediate forward movement and care, are tagged delayed. To determine if a patient is delayed assess the following:

- Does the patient have a peripheral pulse?
- Is the patient not in respiratory distress?
- Is hemorrhage controlled?
- Does the patient follow commands or make purposeful movements?

A "yes" response to **all** of these, but the injuries are still significant, such as a proximal long bone fracture, then the patient should be tagged Delayed.

##### MINIMAL (GREEN TRIAGE TAG)

"Yes" to all of the same questions about pulse, breathing, hemorrhage and mental status, but the patient’s injuries are minor, such as minor abrasions and lacerations, so the patient should be tagged minimal.

### Appendix AG ^§^Scope of Practice

**Note: Information in this appendix may be institution specific. Please evaluate content relevance and insert institute specific information as needed**

#### Objectives

At the end of this activity, the learner will be able to:

1. Understand the need for flexibility between roles during an MCI
2. Understand the golden rule of practice in MCI events
3. Understand MCI Chain of Command and who would make the decision to alter scope of practice

We have an extremely skilled and capable workforce in our ED. Many of our staff members have patient care skills they gained from prior careers or in other jobs they currently hold. We have all sorts of rules by many different entities regarding scope of practice for health care providers. Two examples being:

- Medications paramedics can give in the field or pre-hospital setting but not in the hospital.
- Paramedics and nurses who have been trained as flight nurses, and possibly staff in respiratory therapy who have been trained to intubate patients but that is outside of their scope in the ED.

*To be clear, the authors are NOT condoning, requesting, pressuring or otherwise encouraging any of our staff members to practice outside their defined scope of practice*. However, during an MCI, things may get messy in many ways. Some nimbleness will be required, but some nuances need to be addressed to keep our licenses intact.

**The Golden Rule of Practice in MCI Events: Your scope of practice will not change but your STANDARDS of practice may change.**

If the decision is made to expand the standard of practice, this would come from the incident commander. **Do not “go rogue.”** MCI leadership should anticipate issues so they can request a change from incident command via the established chain of command. The decision would be based on the department need and any needs of patients. The use of altered standards of practice will affect the effectiveness of the response to the MCI. However, there are also legal and financial issues to consider as well.

It is important to remember that the standards of practice may not only vary between MCIs but DURING a single MCI as well. This can be due to several factors; the most significant is making the necessary adjustments to the standards to ensure the care provided in any MCI response results in saving as many lives as possible (refer to Appendix AD^§^ for more information on crisis standard of care). Guidelines when defining standards of practice should consider size, nature and speed of the event, and should also be scalable to the event.

### Appendix AH ^§^Incident Command

**Note: Information in this appendix may be institution specific. Please evaluate content relevance and insert institute specific information as needed**

#### Objectives

At the end of this activity, the learner will be able to:

1. Understand the importance of creating an emergency response structure

**Creating an emergency response structure.** The ED is one small part of the hospital’s response and cannot act independently. An MCI will be chaotic and in order to provide effective response, we will all need to be playing from the same playbook. Department leaders should be appropriately trained in incident command (ICS). ICS is not an emergency response plan, but rather a *structure* that agencies utilize to best respond to disasters. 3 Administration for response will be organized into clearly defined roles with a defined chain of command.

### Appendix AI Email Schedule

[Table t3-jetem-5-3-c1]

**Table t3-jetem-5-3-c1:**

Week	Coordinator	ERT	Provider	RN/Paramedic	Number separate emails
1	Why You Matter	Why You Matter	Why You Matter	Why You Matter	4
2	Getting to the Hospital				1
3	Job Action Cards				1
4	OPEN	^†^Patient Belongings	^§^Crisis Standard of Care		1
5	Decontamination				1
6	Self-care				1
7	Personal Preparedness				1
8	Patient Identification				1
9	Child Management				1
10	OPEN	^†^Reprocessing	^§^Blast injury		2
11	Media Relations				1
12	Other Agencies				1
13	OPEN	^‡^Biological Agents			1
14	OPEN	^†^Trash	^§^SALT triage		2
15	PPE				1
16	Purging the ED				1
17	^*^Coordinator Supplies	OPEN	^§^Scope of Practice		3
18	Disaster carts				1
19	Call out				1
20	^*^Answering Phones	^‡^Airways			2
21	Discharging				1
22	OPEN	^‡^Bleeding Control			1
23	OPEN	^‡^Temporizing for the OR			1
24	OR Prioritization				1
25	OPEN	^‡^Infusing Fluids			1
26	Security				1
27	Evidence preservation				1
28	Family reunification				1
29	Incident Command				1

### Appendix AJ Post-Study Survey Results

This survey was sent to 442 employees, and of those, 54 responded (12%).

Question: Compared to this time last year, which of the following statements is most accurate?

Answer choices:

- I feel more prepared to respond to an MCI compared to last year
- I feel equally prepared to respond to an MCI compared to last year
- I feel less prepared to respond to an MCI now

Response: [Fig f8-jetem-5-3-c1]

Question: Did you prefer long or short emails?

Answer choices:

- Long emails
- Short emails
- No preference

Response: [Fig f9-jetem-5-3-c1]

Below are some comments about preferred email length:

- “I could not possibly have read the lengthy ones. I think I missed a fair amount of content trying to do so. No one really should try to communicate this type of information without bullet points though...so not a surprising outcome” –Senior MD
- “I like shorter emails in general but that said, I read and opened all of them that were sent so the length just made them quicker to get through.” –Resident
- “When e-mails get too long I either don’t have time to read it all or honestly aren’t interested in reading in all the way through.” –RN/medic
- “My attention span is short, I work in an ED!” –RN/medic
- “Would have preferred even shorter--I don’t digest prose well, tend to digest quick bullet points or auditory media (watching videos counts). Additional stimuli were very helpful.” –Senior MD
- “The short emails allow you to open them and be done. When you have 150+ emails every day, there are emails that you can read and file away in your brain and longer emails that you just file away. I DO save the longer emails but rarely get around to going back to them unless there is a need.” –Senior MD

Below are some positive comments about the email project:

- “They were educational and entertaining. “–RN/medic
- “Really useful, concise information” –Senior MD
- “That there was a title like ‘we need supplies’ and it was short and to the point on where we can find disaster carts.” –RN/medic
- “Key information was presented in a humorous way. It was relevant to my role.” – RN/medic
- “Idea of small amounts of info at a time to learn the larger plan” –Resident
- “I liked that they were written informally and sounded like a conversation, was simple language. They are a nice reference to not delete and to have on hand if a question comes up.” –ERT
- “Very educational. I learned so much” –Senior MD
- “I learned things I didn’t know and had a chance to review things I’d forgotten.” –Resident
- “I like having the emails saved and going back to them if I have any questions.” –Coordinator
- “It was an easy way to go over the material instead of reading the entire policy” –RN/medic
- “Thanks for being interested in what makes it interesting and easy to retain important info. This is hard in such a busy department.” –RN/paramedic
- “I really like the emails in general. I think it would be a good idea to make the emails a permanent idea to help refresh the memory of things that we don’t use a lot.” –ERT

Below are some negative comments about the email project:

- “The long emails were too wordy. If a lot of info needs to be distributed maybe try bullet points” –RN/medic
- “Would have preferred an info graphic or 2 min video. “–RN/medic
- “Way too long. Even the short ones. I think ideally this sort of email would be a single sentence. E.g. During an MCI, only the South Entrance will be accessible, including for employees. Maybe maximum 3 sentences. People won’t remember any more details than that.” –Resident
- “I get sooo many emails and I am already on the computer all day for work, and then after work charting, the last thing I want to do on my time off is read work emails.” –PA/PA residents
- “The length of some of them made it hard to really sit down and read them thoroughly before being pulled to another task.” –ERT
- “Not sure how it pertained to my job. Some of the topics seemed irrelevant or not interesting to me.” –RN/medic
- “Some of them were really long” –RN/medic
- “We receive so many emails that it is hard to sift through them all and decide what to read and what not to.” –ERT
- “There is a great deal of information in them and some of it seemed superfluous.” –Coordinator
- “There were a lot of emails but I know there needed to be” –RN/medic
- “Would have liked them to be even shorter.” –Senior MD

Random comments/suggestions:

- “Wasn’t aware that it was happening, and thought it was spam, so deleted them.” –Coordinator
- “Did not recognize the sender. Thought it was spam. Need to do a better job in who is sending the information.” –RN/paramedic
- “Emails are good but do need hands on retraining, and maintaining muscle memory” –RN/medic
